# Supplementary material for: Community matters: Heterogeneous impacts of a sanitation intervention
Source: World Dev. 2023 May;165:106197. doi: 10.1016/j.worlddev.2023.106197 (PMC10008727; doi:10.1016/j.worlddev.2023.106197)
Supplement: Supplementary file 1 [file mmc1.pdf]

Community Matters:

Heterogeneous Impacts of a Sanitation Intervention*

Laura Abramovsky† Britta Augsburg† Melanie Lührmann‡ Francisco Oteiza§
Juan Pablo Rud‡

Sanitation is at the heart of public health policies in most of the developing world, where around 85% of the population still lack access to safe sanitation. We study the effectiveness of a widely adopted participatory community-level information intervention aimed at improving sanitation. Results from a randomized controlled trial, implemented at scale in rural Nigeria, reveal stark heterogeneity in impacts: the intervention has immediate, strong and lasting effects on sanitation practices in less wealthy communities, realized through increased sanitation investments. In contrast, we find no evidence of impacts among wealthier communities. This suggests that a targeted implementation of CLTS may increase its effectiveness in improving sanitation. Our findings can be replicated in other contexts, using microdata from evaluations of similar interventions.

JEL Codes: O12, O18, O13, I12, I15, I18.
Keywords: sanitation, community intervention, randomized controlled trial, Nigeria.

*This research was carried out as part of the Formal Research Component of WaterAid UK's Project 'Sustainable Total Sanitation Nigeria - implementation, learning, research, and influence on practice and policy' (STS Nigeria), funded by the Bill and Melinda Gates Foundation. Abramovsky, Augsburg are also grateful for funding from the ESRC-funded Centre for the Microeconomic Analysis of Public Policy (CPP) at IFS, grant reference ES/T014334/1. The evaluation is registered at the ISRCTN registry (ISRCTN74165567) and received approval from the National Health Research Ethics Committee, Federal Ministry of Health, Nigeria (NHREC/01/01/2007-20-20/11/2014), and the University College London Ethics Committee (2168/009). The authors would like to thank the participants of the EDePo Development Workshop, 3ie seminar, ESPE, Royal Economic Society conference and the London School of Hygiene and Tropical Medicine seminar for helpful comments and suggestions. **Corresponding author:** Britta Augsburg, The Institute for Fiscal Studies, 7 Ridgmount Street, London WC1E 7AE; Phone: 0044(0)20 7291 4800, Fax: 0044(0)20 7323 4780, Email: britta_a@ifs.org.uk. Contacts: Abramovsky (labramovsky@ifs.org.uk), Lührmann (melanie.luhrmann@rhul.ac.uk), Oteiza (foa@osloeconomics.no), Rud (Juan.Rud@rhul.ac.uk). The authors declare that they have no known competing financial interests or personal relationships that could have appeared to influence the work reported in this paper.

†The Institute for Fiscal Studies
‡Department of Economics, Royal Holloway, and IFS
§Oslo Economics

1 Introduction

Public health interventions are often promoted as drivers of behavioral change and catalysts in the investment and adoption of health technologies. The introduction of comprehensive water and sanitation programs in developed nations in the early 1900s has been dubbed the most effective public health intervention of the last century (Alsan and Goldin, 2019), partly because of the significant cost of poor sanitation and the disease environment it creates. These costs include negative impacts on child health (Alzúa et al., 2020, Augsburg and Rodriguez-Lesmes, 2018, Baird et al., 2016), morbidity (Prüss-Ustün et al., 2014), human capital other than health (Adukia, 2017, Orgill-Meyer and Pattanayak, 2020, Spears and Lamba, 2016), and psycho-social stress (Sahoo et al., 2015). Such programs can provide the foundation for economic growth. Currently 3.6 billion people still lack access to safely managed sanitation (WHO/UNICEF, 2021), most of whom live in low and middle income countries (LMICs). Improving access to sanitation has thus been recognized as a key goal towards sustainable development by the United Nations.

However, the effectiveness of public health interventions, including sanitation ones, in LMICs is little understood, especially when implemented at scale (Banerjee et al., 2017, Cohen and Dupas, 2010, Dupas, 2011). We address this knowledge gap by evaluating a participatory community-level intervention, Community-Led Total Sanitation (CLTS), which aims at improving access to safe sanitation and has been rolled out by more than 25 governments around the world (Zuin et al., 2019). CLTS entails community meetings and the provision of information with the aim of eradicating open defecation (OD) by triggering collective behavioral change and encouraging communities to construct and use toilets. While the approach has been evaluated in the past, divergent and inconclusive results suggest that the circumstances under which CLTS is an effective tool to improve sanitation in LMICs are not well understood (Radin et al., 2020), preventing efficient targeting (Brown et al., 2019).

In this paper, we use a cluster randomized controlled trial (RCT) to study the effectiveness of CLTS implemented at scale in Nigeria. CLTS is the main pillar of the Government of Nigeria's 'National Strategy for Scaling up Sanitation and Hygiene'. The study comprises 247 study clusters of rural communities. We randomly selected 20 households per cluster for interview at baseline in

2014 and conducted three follow-up surveys 8, 24 and 32 months after CLTS implementation to study its short- and longer-run impacts on OD and sanitation investments. Low attrition allows us to work with a balanced panel of more than 4,500 households, located in the Nigerian states Ekiti and Enugu.

Nigeria is a particularly apt context to study sanitation policies. Accounting for about half of West Africa's population, it is a key player in the region. Yet, the country is facing massive developmental challenges, evidenced for example by its ranking of 152 out of 157 countries in the World Bank's 2018 Human Capital Index (World Bank, 2019). In 2020, almost 60% of its 206 million strong population had no (18.7%), only limited (19.5%) or unimproved (19.1%) access to sanitation in 2020, and these rates have only marginally improved since the early 2000s. In consequence, Nigeria is a top contributor to the share of the global population without access to adequate sanitation (WHO/UNICEF, 2021).

We show that, on average, CLTS led to small and temporary reductions in OD among households in treated communities, i.e. a reduction of 3 percentage points (ppt) that was not sustained 32 months after the baseline. However, these estimates hide strongly varying impacts across population subgroups: we find that intervention impacts are considerably stronger among, and restricted to, the asset-poorest half of the studied communities. In these less wealthy communities, households constructed toilets, leading to a 9 ppt drop in OD, three times as much as on average. This effect was first measured after 8 months, and was sustained over the whole 32-month study period. It is substantial and comparable to interventions that also include a financial component. The average impacts of sanitation interventions that combine CLTS with subsidies or financial incentives for toilet construction range between 9 and 19 ppts (Andres et al., 2020, Guiteras et al., 2015, Patil et al., 2014). In addition, our results for CLTS in less wealthy communities are comparable to the impact of the provision of sanitation-tagged microcredit in India (Augsburg et al., 2021a), which resulted in a 10 ppt reduction of OD.

The finding that CLTS interacts with community characteristics links our study to the theory of geographical poverty traps which puts forward that different neighborhood endowments (of physical and human capital) may lead to different outcomes for otherwise identical households. Jalan and

Ravallion (2002) argue that, in consequence, interventions have a trajectory that depends on the neighborhood even when all household characteristics are accounted for. They further argue that targeting of geographical areas may be easier and more effective given unconstrained mobility and limited data to identify poor households. We show (in the spirit of Ravallion and Wodon (1999)) that CLTS induces wealthier and less wealthy households to invest in sanitation when they live in less wealthy communities, while we do not find CLTS impacts on asset-poor or asset-rich households living in wealthier communities. We further show that community wealth encompasses a number of distinctive community characteristics that are already known to correlate positively with sanitation investments (such as pre-treatment differences in toilet ownership, measures of social cohesion, or leader characteristics). None of these characteristics could independently account for the strong impacts of CLTS in less wealthy communities, but they may interact to produce the stronger CLTS impacts in less wealthy communities. This leaves us with one very robust predictor of CLTS effectiveness: community wealth.

We use this result to propose a simple strategy to target CLTS. Measures of wealth are readily available in standard household surveys. Alternatively, wealth proxies such as nightlight intensity indices can be obtained from open access satellite data. The Government of Nigeria can use these data to develop a national targeting strategy. We illustrate this based on the 2013 Nigerian Demographic and Health Survey (DHS), and show that the data can replicate the classification of geographical areas into less wealthy and (modestly) wealthier ones with a precision that is similar to the (more detailed) measures obtained from our primary study data.

Finally, we conduct an exploratory analysis to gain insights into whether community, or area, wealth could also be used as a basis for targeting CLTS implementation in other contexts. We pool microdata from Indonesia, Nigeria and Tanzania and estimate average and heterogeneous CLTS impacts on toilet ownership along nightlight intensity – a globally available proxy for community wealth that is comparable across contexts. We find an inverse relationship between community wealth and CLTS impacts, further supporting the conjecture that area-level wealth is a plausible underlying factor of CLTS program effectiveness beyond our Nigerian RCT. The results rationalize the wide range of CLTS impacts documented in the literature.

In summary, our findings allow us to draw policy-relevant conclusions regarding a wider roll-out (Muralidharan and Niehaus, 2017), especially since they are based on implementation at scale. CLTS can be an effective policy tool if appropriately targeted. We also show it closes the sanitation gap between less and more wealthy communities. Yet, despite its popularity and wide implementation, CLTS needs to be complemented with other interventions to close the large sanitation gap remaining overall, as more than a third of households in wealthier communities and more than half of households in less wealthy communities continue to openly defecate after implementation of the intervention.

The remainder of the paper is structured as follows. The next section describes the intervention. Section 3 presents the experimental design and Section 4 describes the data collection and balance in the randomisation. Section 5 presents the empirical method and Section 6 our impact estimates. Section 7 lays out our proposed targeting strategy and compares the results of our study with those of other CLTS interventions. Section 8 concludes.

2 The intervention

Our study focus is two of three states where the international non-governmental organization (NGO) WaterAid worked closely with local governments and local NGOs in implementing CLTS with a view to improving toilet coverage and reducing open defecation. In particular, WaterAid Nigeria and two local NGOs active in the study states Ekiti and Enugu[1], trained local government authority (LGA)[2] staff in water, sanitation and hygiene (WASH) units, which are part of Nigeria's public service. WASH officials then took responsibility for CLTS delivery and implementation. In the context of the evaluation study, mobilization and triggering activities took place over six months – between January and June 2015.

The implementation followed the three-step CLTS approach. First, community leaders are engaged in a discussion about the negative health implications of OD,[3] as well as the potential

[1] The 2014 National Nutrition and Health Survey revealed that just 29% of households in Enugu and 46% of households in Ekiti had access to improved sanitation. This resulted in rates of OD of 51% and 44% respectively, according to the same survey.

[2] LGAs are Nigeria's second administrative sub-division, led by a local government council.

[3] A further key message is the importance of sanitation externalities, i.e. that all community members (particularly

benefits of CLTS in achieving behavioral change within their communities. Community leaders then arrange a community meeting, the so-called 'triggering meeting', the main component of CLTS. The meeting starts once and only if a significant number of community members gathered in a predefined public space on the appointed day. The first activity is a community mapping exercise, in which each attending community member marks their household's location and toilet ownership status on a stylized map on the ground. Community members next identify and mark regular OD sites. In many cases, facilitators follow up with graphic images showing that the community lives in an environment contaminated by feces. Facilitators of the meetings further use the map to trace the community's contamination paths of human feces into water supplies and food.[4]

As a closing task, attendees are asked to draw up a community action plan to achieve community-level open-defecation-free (ODF) status to foster collective action and collaboration. The action plan includes discussions of how vulnerable or less wealthy households can be supported to achieve ODF status. It is posted in a public spot. Volunteers (so-called 'natural leaders') are chosen to follow up regularly on each attendee's commitment towards implementing the plan. They hence carry the main responsibility for follow-up, but without any formal authority to push for action. CLTS facilitators were asked to conduct one follow-up visit to communities, checking in with natural leaders on communities' advances to become open defecation free. Eventually, the community can be certified for its achievements by the national Rural Water Supply and Sanitation Agency (RUASSA) and the National Task Group on Sanitation (NTGS).

CLTS does not offer any monetary incentives, subsidies or credit to finance toilet construction or reward OD reductions or ODF achievement. It also does not provide technical assistance or hardware nor does it promote a particular toilet technology. CLTS is expected to drive a change in sanitation practices purely by altering the perceived costs of unsafe sanitation and the perceived benefits of toilet use.

children) are at risk of contracting sanitation-related diseases if some residents practice open defecation.

[4] Other activities may be added at the discretion of the facilitator. Examples include medical expense calculations related to illnesses likely induced by OD practices; transect walks through the community (often referred to as 'walks of shame'), pointing out visible feces in the environment to evoke disgust and shame; or graphic exercises, where facilitators might add feces to drinking water, illustrating that these are not necessarily visible to the naked eye. In the context of our study, about 20% of triggering meetings included at least one such additional exercise. For example, 14% of triggering meetings included graphic illustration and 7% conducted expense calculations.

3 Research design

The research design is a two-stage cluster randomized controlled trial. In the first stage, the unit of interest is communities, in line with CLTS being a community-level intervention. In the second stage, the unit of interest is residents of these communities. Our sample is representative of the study area, consisting of 246 communities in nine LGAs in Ekiti and Enugu, which contrasts with sanitation evaluations that often focus on households with children as, for example, in Cameron et al. (2019) and Pickering et al. (2015).

Communities (clusters) in this study do not match Nigeria's administrative units. Rather, they were defined closely with local implementing partners to capture adequate implementation clusters and reduce information spillover, i.e. as self-contained units so that information about triggering activities would not spread to the next cluster, for example via shared markets or large public areas. To further safeguard the validity of the stable unit treatment value assumption (SUTVA), 'buffer' areas were introduced to ensure that no two clusters were located in close geographic proximity. A community comprises on average 1.7 villages or quarters,[5] where CLTS was implemented at the same time.

In total, 246 communities were randomized with equal probability into either receiving CLTS (treatment) or not receiving it during the course of the study (control). Randomization was stratified by LGA.[6] The distribution of treatment and control clusters is presented in Online Appendix Table A1 and the location of study communities is indicated in Online Appendix Figure A1.

Within these communities, we conducted a resident census during October 2014. The census covered basic information from 50,333 households (27,888 in Enugu and 22,445 in Ekiti) in the participating LGAs, and served as our household sampling frame. More details on the research design are provided in Abramovsky et al. (2015).

[5] Village is the term used in the state of Enugu and quarter in Ekiti. The median and modal number of villages or quarters within a cluster are both 1. The maximum number of villages in a cluster is 7, occurring only once.

[6] Study LGAs in Enugu are Igbo Eze North, Igbo Eze South, Nkanu East and Udenu. In Ekiti, Ido Osi, Ikole, Moba, Irepodun Ifelodun and Ekiti South West are part of the study.

4 Data

We collected panel information on communities and households within these communities at four points in time over a period of 32 months.[7] The baseline survey, administered to 4,540 households, took place during December 2014 and January 2015. To follow the behavior of community residents over time, three follow-up surveys were conducted – after 8 months (FU1: Dec 2015 to Feb 2016), 24 months (FU2: March to April 2017) and 32 months (FU3 or 'endline survey': Nov 2017 to Jan 2018); see timeline in Online Appendix Figure A2. The three post-intervention surveys allow us to study the sustainability of CLTS impacts over time up to about three years. Household attrition rates over the three follow-up survey rounds are low: 2.53% at FU1, 8.81% at FU2 and 11.58% at FU3. There is no differential attrition across experimental groups (see bottom panel of Table 1 and Online Appendix C).

Households in study communities are typically headed by a male (64.5%) with at least primary education (67.5%), who is employed (78.1%), mostly in farming (47.1%) and on average 56 years old. Households consist of 4 members on average, and almost a third have at least one child under the age of 6 years (Table 1). These characteristics are balanced across experimental arms along a set of 22 indicators, except for a small (0.27) difference in the number of household members, which we hence include as a covariate throughout our analysis.[8]

Almost two thirds of households (62.8%) have at least one member above the age of 4 years defecating in the open. A similar percentage of main respondents (62.4%) report to openly defecate themselves.[9] Prevalence of open defecation in our study population is closely aligned to that of our study states Ekiti and Enugu, as reported in the 2015 Nigeria Malaria Indicator Survey (NMIS) at 64.75%. In line with these behavioral measures, only 36.9% of households own a toilet, 36.1% own a

[7] Data collection was carried out by an independent data collection company, blinded to treatment status. The survey instruments were developed jointly with local implementing partners knowledgeable about the study areas, were piloted in the field and were further adjusted after interviewer training. These processes were followed to ensure respondents would understand, and feel comfortable with, all questions asked.

[8] In an F-test of joint significance of all characteristics, we reject the null hypothesis at the 5% level (p-value=0.038). Yet, once we remove household size, the explanatory power of the remaining variables falls markedly (p-value=0.27), supporting the validity of our randomization strategy across all other dimensions of household and community measures. Abramovsky et al. (2015) report balancedness of our randomization on an even wider set of characteristics.

[9] Both rely on the question 'Where do you [each family member] go to defecate [most frequently]?', combined with a showcard of possible places. Urination habits are asked separately at baseline.

functioning toilet of any type, and 32.4% own a functioning and improved toilet at baseline; 98% of households that have a toilet use it at baseline. These toilet ownership measures, which integrate results from interviewer inspections, capture different dimensions of interest. The first records ownership, but ignores functionality. The second (functional toilet ownership) additionally captures whether maintenance investments into the existing stock of toilets are made. The third measure (functional improved toilet) accounts for quality beyond functionality, satisfying the stricter criteria set by the WHO/UNICEF Joint Monitoring Program regarding improved sanitation.

Characteristics of the study communities in which these households reside are shown in the third panel of Table 1. They include an aggregated indicator of households' wealth to capture community wealth. The index is measured as the first factor of a principal component analysis based on a series of questions regarding asset ownership.[10] While its numerical value is not meaningful in itself, comparing it with the distribution of community wealth across Nigeria using DHS 2013 data (see Online Appendix F for details), we find that communities in our sample are typically located towards the middle (4th to 7th deciles) of the Nigerian wealth distribution, rather than in the tails. Hence, our sample communities are neither very asset-rich nor very asset-poor in terms of wealth relative to the Nigerian distribution. Our analysis will particularly focus on heterogeneity in CLTS effectiveness by this community-level wealth measure, as well as an alternative proxy, pre-intervention nightlight intensity within a 5km radius.[11] We find that average night light intensity in our study area is very low with a mean of 2 relative to the global night light range of 0 to 63.

The next set of community characteristics listed in Table 1 relate to social interactions within the community, suggested as accelerators of the effectiveness of CLTS (see, for example, Cameron et al. (2019)): a community's level of (i) trust, (ii) community participation, and (iii) religious fragmentation. Trust is the average community score of the degree to which its members trust their neighbors. Community participation is constructed similarly, based on households' participation in community events. Religious fragmentation is adapted from measures used in studies of ethno-linguistic fragmentation (ELF), as our study sample is homogeneous along ethnic lines but very diverse in terms of religion. Finally, mean toilet ownership rate per community is 36.2%. Detailed

[10] Details of its components and their factor loadings are provided in Online Appendix Table B1.
[11] Michalopoulos (2013) presents evidence that wealth and night light intensity are strongly correlated.

Table 1
Balance between treatment and control groups at baseline

	All	Control			Treatment-Control	
	Obs.	Obs.	Mean	SD	Coeff.	*p*-value
Panel A - Post-attrition household sample						
Household characteristics						
Head male (%)	4,014	2,027	64.53	47.85	-1.92	0.307
Head age (years)	4,014	2,027	55.82	17.23	-0.66	0.364
Head employed (%)	4,014	2,027	78.10	41.37	-0.79	0.677
Head finished primary school (%)	4,014	2,027	67.54	46.83	0.40	0.851
Household size	4,014	2,027	4.33	2.50	-0.27	0.022**
Household has at least 1 child below 6 y/o (%)	4,014	2,027	30.64	46.11	-0.49	0.792
Primary activity is farming (%)	4,014	2,027	47.11	49.93	3.06	0.414
Asset wealth index score	4,014	2,027	0.05	2.04	-0.02	0.879
Open defecation and toilet ownership						
At least 1 member (> 4 y/o) performs OD (%)	4,014	2,027	62.80	48.35	0.66	0.838
Main respondent performs OD (%)	3,974	2,008	62.40	48.45	0.47	0.884
Own a toilet (any condition, any type) (%)	4,014	2,027	36.90	48.27	-0.31	0.922
Own a functioning toilet (any type) (%)	4,014	2,027	36.11	48.04	-0.18	0.955
Own a functioning, improved toilet (%)	4,014	2,027	32.36	46.80	0.40	0.896
All household members use a toilet (%)	4,014	2,027	33.45	47.19	0.37	0.903
All household members use a toilet (cond. on ownership) (%)	1,446	732	92.62	26.16	1.49	0.327
Panel B - Community characteristics						
Community wealth	245	121	-0.21	0.95	-0.13	0.353
Night light intensity, 5km radius, 2013 (min = 0, max = 25)	245	121	2.07	3.01	-0.06	0.870
Trust in neighbors (0=none, 2=high)	245	121	0.88	0.49	0.01	0.847
Community Participation Index	245	121	-0.09	1.10	0.16	0.252
Religious fragmentation (0=low, 1=high)	245	121	0.61	0.16	0.02	0.465
Toilet ownership rate (%)	245	121	36.24	24.51	-1.32	0.674
Panel C - Attrition						
Not surveyed at endline (%)	4,540	2,281	11.14	31.46	0.91	0.341

Notes: Data from baseline household survey. Panel A: sample includes only households also surveyed at endline. Panel B: reports statistics for study communities. Panel C: sample includes all households surveyed at baseline.
'Improved toilets' refers to toilets of the quality defined using the classification in WHO/UNICEF (2021). For a detailed description of household and community-level covariates, see Online Appendix B. **p* <0.10, ** *p* <0.05, *** *p* <0.01.

definitions of these measures and their distribution can be found in Online Appendix Section B.2.

5 Estimation approach

Following successful randomization evidenced in Table 1, we estimate the impact of CLTS on our primary outcome, open defecation practices, using an intent-to-treat (ITT) design based on cluster randomized assignment to treatment.[12] We compare open defecation practices y_{ict} in household i living in community (cluster) c in period t by treatment assignment:

$$y_{ict} = \alpha + \gamma T_c + X_{ic0}\beta + \theta y_{ic0} + \omega_g + \delta_t + \epsilon_{ict} \tag{1}$$

where community-level CLTS treatment status is defined by T_c. Baseline characteristics of households and their heads, X_{ic0}, are included alongside LGA and survey wave fixed effects, ω_g and δ_t, to control for unbalanced household size, unobserved area effects and contemporaneous shocks and to increase the precision of our estimates. The parameter of interest, γ, captures the average impact of CLTS. Our preferred analysis of covariance (ANCOVA) specification further conditions on the baseline value of the outcome variable, y_{ic0}. These estimates are more efficient than difference-in-difference and simple difference estimators in experimental contexts, when pre-treatment information is available and the outcome is strongly correlated over time (McKenzie, 2012). Alongside, we present conventional difference-in difference (DiD) estimates.

We investigate heterogeneous impacts, primarily along community characteristics (CCs), in the expanded specification:

$$y_{ict} = \alpha + \gamma_r T_c + \gamma_d (T_c \times CC_c) + \phi CC_c + X_{ic0}\beta + \theta y_{ic0} + \omega_g + \delta_t + \epsilon_{ict} \tag{2}$$

where we introduce a binary variable CC_c, and include the interaction term $T_c \times CC_c$. In our main results, we define CC_c as low and high community wealth, split along the sample median. The γ_r parameter captures the average CLTS treatment effect in the less wealthy half of communities (for which $CC_c = 0$), and γ_d is the difference in treatment effects between communities with above and

[12] In a successfully randomized scenario, as is our study (see Table 1), ITT designs yield unbiased estimates of the average impact of the intervention on the sample assigned to treatment. Additionally, we follow Imbens and Angrist (1994) and Angrist and Imbens (1995) and instrument triggered treatment with treatment assignment in Section 6.2. The results are very similar to the ITT estimates. In Online Appendix C we show that there is no evidence of selective triggering in our study.

below median wealth.

Since we are testing multiple hypotheses simultaneously in our analysis of heterogeneous impacts, we report both, unadjusted (or naive) p-values and p-values that are adjusted for the family-wise error rate in brackets. We compute the latter using the methodology proposed by Romano and Wolf (2005), calculated by drawing 1,000 clustered bootstrapped samples.

6 Results

Table 2 presents estimates of average treatment effects on open defecation behavior, defined as a dummy equal to 1 if the main respondent performs OD, 0 otherwise. Columns 1 and 2 show the simple DiD estimates with and without covariates, and Column 3 presents ANCOVA results. We find that CLTS reduced OD consistently across all specifications when we pool observations across the three follow-up surveys (Panel A), with ANCOVA providing the highest precision (p-value<0.05). However, the magnitude of behavioral change is small – exposure to CLTS resulted in a reduction in OD by 4 ppts eight months after CLTS. This reduction is sustained for two years after intervention implementation, but then fades out (Panel B). We find no evidence of systematic measurement error in the treatment group as a result of CLTS, due to over-reporting of 'desirable' outcomes, for example: only 0.5% of households in the control and 0.9% in the treatment group report non-existing toilets at endline, and the difference is not statistically significant (p-value=0.115). Our results are also robust to using an alternative measure of OD, namely whether at least one household member above the age of 4 performs OD (Online Appendix D).

This effect size is towards the lower bound of the range of impact estimates found in other evaluations of CLTS. CLTS evaluations in Indonesia (Cameron et al., 2019) and Bangladesh (Guiteras et al., 2015) found no statistically detectable reductions in OD. On the other hand, other studies have shown that CLTS can change behavior, including in Tanzania (Briceno et al., 2017) and India (Pattanayak et al., 2009), with reductions in OD as high as 30 ppts in Mali (Pickering et al., 2015).[13] A recent cross-country study concludes that '[t]he impact of CLTS and subsequent

[13] Pattanayak et al. (2009) was a community-led intervention in the context of subsidy provision, which the authors state were hardly availed though. Other RCT-based evidence is available for CLTS, but these studies evaluate CLTS bundled with other interventions. E.g. Guiteras et al. (2015) include a treatment arm where the CLTS-like

Table 2
CLTS impacts on open defecation

Dep. variable: Main respondent performs OD	DiD		ANCOVA
	(1)	(2)	(3)
Panel A - Pooled impacts			
CLTS (γ)	-0.03	-0.04	-0.03**
	(0.22)	(0.14)	(0.04)
Panel B - Impacts over time			
CLTS x FU1	-0.04	-0.04	-0.04**
	(0.16)	(0.11)	(0.04)
CLTS x FU2	-0.03	-0.04	-0.03*
	(0.23)	(0.14)	(0.09)
CLTS x FU3	-0.02	-0.03	-0.03
	(0.42)	(0.34)	(0.22)
Household controls	No	Yes	Yes
Control mean	0.48	0.48	0.48
Communities	246	246	246
Observations	13,233	12,830	12,697

Notes: Estimates based on OLS regression using equation 1. Panel A reports estimates using data pooled across all three post-intervention survey waves, while Panel B shows estimates by follow-up survey waves where FU 1, 2 and 3 denote measurements from waves conducted 8 (FU1), 24 (FU2) and 32 months (FU3) after baseline. DiD (ANCOVA) refers estimates obtained using a difference-in difference (ANCOVA) estimator. Household controls are: age, gender, education attainment and employment status of the household head; household size, whether the household has at least one child below age 6, household wealth asset score, and whether farming is the household's main economic activity. Standard errors are clustered at the community level. p-values are shown in parentheses. *$p<0.10$, ** $p<0.05$, *** $p<0.01$.

sustained latrine use varied more by region than by intervention, indicating that context may be as or more important than the implementation approach in determining effectiveness' (Crocker et al., 2017). In consequence, we focus on the estimation of heterogeneous impacts across communities in the remainder of the paper.

6.1 Heterogeneous impacts across communities

CLTS is designed and implemented as a participatory intervention at the community level, with the aim of bringing about collective change. In spite of its current popularity, the available evidence does not provide clear guidance for successful targeting, which requires a better understanding of

intervention is coupled with subsidies, and another with subsidies and a supply side technical assistance intervention. The World Bank carried out two further RCTs on the effectiveness of CLTS in combination with subsidy provision in India (Patil et al. (2014), Andres et al. (2020)).

the characteristics that best predict the effectiveness of CLTS.

The pioneers of the approach argue that the impact of CLTS on sanitation outcomes may vary by the socio-economic status of treated communities (Kar and Chambers, 2008). Following this hypothesis, we use community wealth as a widely available, comprehensive proxy for local socio-economic status (SES) and investigate heterogeneous CLTS impacts along this dimension.[14] We discretize community wealth along the sample median by ranking communities according to their wealth score. Communities with wealth scores equal to or above the median are defined as 'high-wealth' communities ($CC_c = 0$), while the rest are classified as 'low-wealth' communities ($CC_c = 1$). Comparison with the Nigerian wealth distribution suggests that a more accurate labeling would be lower- and upper-middle wealth groups (see Online Appendix Figure F3).

Using the pooled sample, we indeed find strong heterogeneity in impact estimates by community wealth. Table 3 shows that CLTS reduced OD prevalence by 9 ppts in less wealthy communities (γ_r from Equation 2). The difference from wealthier communities is also highly significant and almost equal in magnitude, implying statistically insignificant impact estimates close to zero in wealthier communities.

A split of communities by wealth quartiles confirms this heterogeneity: Figure 1 shows that CLTS impacts are largest in the first quartile, statistically significantly different from zero up to median wealth, and not different from zero among higher quartiles.[15] This supports the median split presented in Table 3 to identify communities with positive CLTS impacts, but additionally points to non-linear, even stronger impacts in the first quartile, i.e. among the least wealthy communities.

The OD reductions in less wealthy communities, achieved through CLTS implementation, are driven by increased sanitation infrastructure investment (Columns 2 to 5 of Table 3). OD reductions are almost identically matched by an increase in toilet ownership of 8 ppts (Column 2). Ownership of *functioning* toilets (Column 3) increased by 10 ppts, suggesting that 2% of existing toilets were kept functional due to CLTS. In contrast, we do not find that CLTS triggered higher usage

[14] Detailed lists of household asset items are frequently elicited in household surveys in developing countries, as they are often more precise than measures of household income. The aggregated index has mean 0 and a standard deviation of 1. See details in Online Appendix Section B.

[15] Our results are also qualitatively and quantitatively robust to using a continuous specification of community wealth (see Online Appendix Section E.3).

TABLE 3
Community wealth-specific CLTS impacts on OD and sanitation investments

Outcome =1 if	Performs OD	Owns toilet	Owns functioning toilet	Uses toilet (if functioning)	Shares toilet with neighbors
	(1)	(2)	(3)	(4)	(5)
CLTS x low wealth	-0.09***	0.08***	0.10***	0.03	-0.00
p-value (naive)	(0.00)	(0.00)	(0.00)	(0.17)	(0.95)
p-value (FWE corrected)	[0.03]	[0.05]	[0.01]	[0.46]	[0.96]
Difference	0.10***	-0.11***	-0.12***	-0.05	0.02
p-value (naive)	(0.00)	(0.00)	(0.00)	(0.13)	(0.32)
p-value (FWE corrected)	[0.05]	[0.03]	[0.01]	[0.43]	[0.51]
Control mean (high wealth)	0.37	0.74	0.62	0.64	0.06
Control mean (low wealth)	0.60	0.53	0.40	0.68	0.07
Communities	246	246	246	211	246
Observations	12,697	12,497	12,497	2,548	12,697

Notes: 'Difference' is the coefficient γ_d from estimating equation 2, indicating the difference in treatment effects between communities with above and below median wealth. Control means are calculated using endline data. Household controls are: age, gender, education attainment and employment status of the household head; household size; whether the household has at least one child below age 6; household wealth asset score; and whether farming is the household's main economic activity. Standard errors are clustered at the community level. Naive (unadjusted) p-values are shown in parentheses. In brackets we present p-values adjusted by family-wise error (FWE) rate following Romano and Wolf (2005), using 1,000 cluster bootstrap samples. **p <0.10, ** p <0.05, *** p <0.01.*

of existing toilets, shared or otherwise (Columns 4 and 5). This is likely driven by the fact that reported (and interviewer-observed) usage of owned toilets at baseline was already close to universal (Table 1), and usage rates of newly constructed toilets remain high.[16] These findings are in line with ownership and use of private toilets being the most frequently discussed channels to reduce OD in CLTS community meetings (Kar, 2003).

Figure 2 shows that the impacts of CLTS on OD (left panel) and functioning toilet ownership (right panel) in less wealthy communities are achieved within 8 months, and are sustained across the three follow-up periods spanning 32 months in total, suggesting that the initial triggering worked akin to a one-shot policy in the Nigerian context. Orgill-Meyer et al. (2019) find similarly persistent effects on toilet ownership in the context of India even over a 10 year period, but – contrary to our setting – toilet ownership and OD practice diverged early on, and OD reductions could only be established one year after the intervention.

[16] The percentage of (functioning) toilet owners who report that their household does not practice OD remains very similar after the intervention (95%), and does not differ between households in treatment and control groups (p-value=0.345). This is different from OD habits in India, for example, where toilet ownership and usage do not necessarily go hand in hand (see, for example, Gupta et al. (2020)).

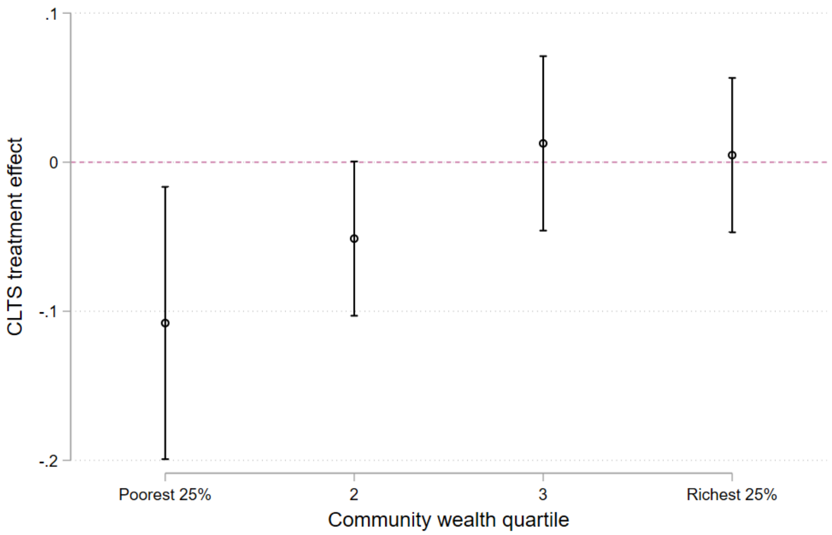

FIGURE 1
CLTS impacts on OD by community wealth quartile

Notes: The figure displays estimated confidence intervals for CLTS treatment effects by community wealth quartiles. Household controls are: age, gender, education attainment and employment status of the household head; household size; whether the household has at least one child below age 6; household wealth asset score; and whether farming is the household's main economic activity. Standard errors are clustered at the community level.

6.2 Exploring alternative margins for CLTS impacts

In this section we explore whether other community and individual characteristics that might be correlated with community wealth may be more policy-relevant margins for CLTS impacts or better predictors of its effectiveness.

Is impact heterogeneity driven by community differences in compliance?

An immediate concern could be that CLTS may be ineffective in wealthier communities due to lower compliance, i.e. a lower likelihood of triggering meetings taking place. Yet, we do not find evidence of statistically significant differences in compliance (p-value=0.301). We nonetheless present treatment-on-the-treated estimates in Table 4, obtained through an instrumental variable (IV) strategy. Our treatment indicator T_c becomes 1 if triggering activities actually took place, and the instrumental variable is the assignment to triggering, following Imbens and Angrist (1994) and Angrist and Imbens (1995). We find qualitatively and quantitatively very similar results

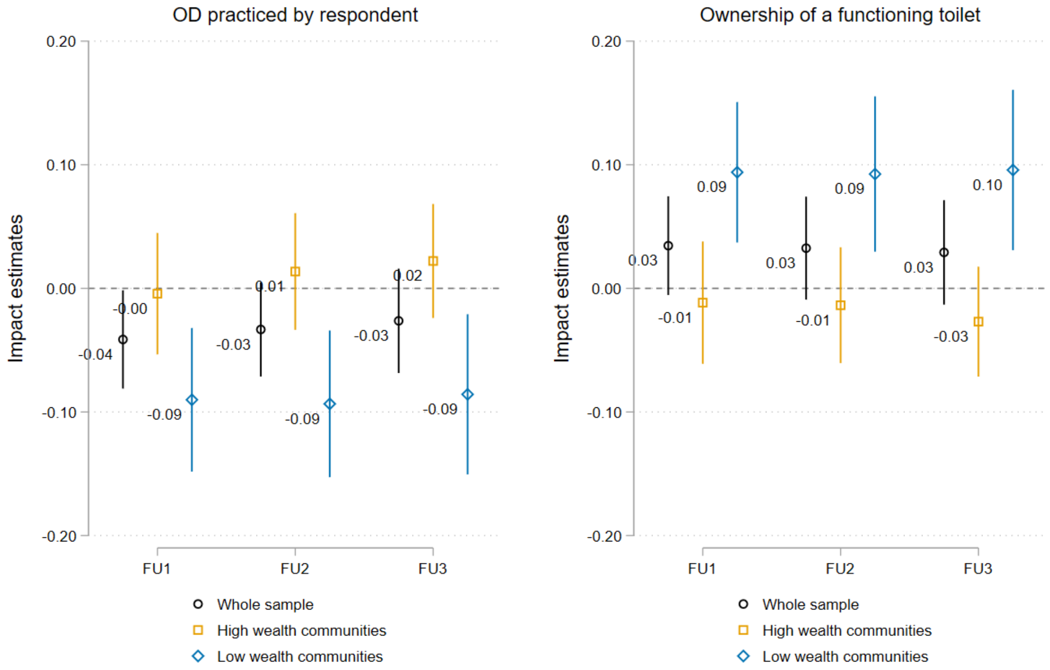

FIGURE 2

Dynamics of CLTS treatment effects on OD and toilet ownership

Notes: Graphs plot the point estimates and 95% confidence intervals for CLTS impacts on OD (left) and toilet ownership (right) by follow-up survey wave and community wealth. FU 1, 2 and 3 refer to measurements obtained from followup survey waves conducted 8, 24 and 32 months after baseline. Household controls are: age, gender, education attainment and employment status of the household head; household size; whether the household has at least one child below age 6; household wealth asset score; and whether farming is the household's main economic activity. Robust standard errors are clustered at the community level.

compared with the ITT estimates: CLTS is only effective in triggered communities with wealth below the median, where OD is reduced by 10ppts. It is ineffective in triggered communities that are wealthier.[17]

TABLE 4
CLTS impacts by triggering status, IV estimates

Dep.variable: main respondent performs OD			
	All	Wealthier communities	Less wealthy communities
CLTS	-0.04**	0.02	-0.10***
	(0.04)	(0.54)	(0.00)
First-stage F-statistic	641	172	820
Communities	246	123	123
Observations	12,697	6,515	6,182

Notes: The instrumental variable is the initial, randomized treatment assignment of the community based on the census. 'Triggering' denotes whether the triggering activities defined in Section 2 actually took place.
Household controls are: age, gender, education attainment and employment status of the household head; household size; whether the household has at least one child below age 6; household wealth asset score; and whether farming is the household's main economic activity. Standard errors are clustered at the community level. Naive (unadjusted) *p*-values are shown in parentheses. **p <0.10, ** p <0.05, *** p <0.01.*

Is impact heterogeneity driven by lower baseline toilet ownership in less wealthy communities?

Furthermore, wealth is positively correlated with baseline toilet coverage, and therefore less wealthy communities may have a higher adjustment margin to react to CLTS. To understand whether wealth simply picks up low initial toilet ownership, we estimate heterogeneous impacts by baseline toilet coverage instead of community wealth. CLTS impacts on OD are 4 ppts larger in areas with low initial toilet coverage (Column 1 in Table 5), but not significantly different from zero and in economic magnitude considerably smaller than the heterogeneous community wealth impacts. This suggests that community wealth is a more informative measure for CLTS effectiveness than toilet coverage. On the other hand, when considering night light intensity, which also correlates strongly

[17] The shares of communities assigned to CLTS in which triggering meetings were successfully run were similar (83% in less and 75% in more wealthy communities), and not significantly different (*p*-value=0.301). Attendance rates at the triggering meeting, measured as the number of attendees recorded by CLTS facilitators over village population, were also not significantly different between more (34%) and less wealthy communities (42%). A community level regression of attendance rates on community level wealth group (i.e. a dummy equal to 1 if the community is less wealthy) and LGA fixed effects yields a point estimate of just 3ppts and a *p*-value of 0.662.

with wealth and has been used in the past to proxy for GDP per capita, a measure of income, at the sub-national level in African countries (Michalopoulos, 2013) we find similar patterns as with asset wealth. Areas characterised by low nightlight intensity show significant reductions in OD due to CLTS, and these are significantly different to impacts in areas that experience high night light intensity. The difference becomes, however, insignificant when considering adjusted p-values.

TABLE 5
Sensitivity analysis: impact heterogeneity by baseline toilet coverage, night light intensity and individual wealth heterogeneity

Dep.variable: main respondent performs OD<br>Community characteristic<br>at baseline/sample	Impacts by community trait		Impacts by household wealth		
	(1)	(2)	(3)	(4)	(5)
	Toilet coverage	Night light	Whole sample	Wealthier communities	Less wealthy communities
CLTS x Low	-0.05*	-0.07***	-0.06**	0.01	-0.11***
p-value (naive)	(0.08)	(0.01)	(0.01)	(0.76)	(0.00)
p-value (FWE corrected)	[0.32]	[0.06]	[0.10]	[0.93]	[0.01]
Difference	0.04	0.08**	0.05**	0.00	0.05
p-value (naive)	(0.25)	(0.02)	(0.03)	(0.87)	(0.17)
p-value (FWE corrected)	[0.56]	[0.16]	[0.18]	[0.93]	[0.52]
Control mean (Low)	0.66	0.57	0.60	0.45	0.69
Control mean (High)	0.34	0.39	0.37	0.33	0.47
Communities	246	246	246	123	123
Observations	12,697	12,697	12,697	6,515	6,182

Notes: 'Difference' is the coefficient γ_d from estimating equation 2. Column 1 shows impact estimates for communities with baseline toilet coverage above and below the median. In Column 2, we present estimates using night light intensity above and below the median as an alternative measure of community wealth. Columns 3 to 5 present CLTS impact estimates for *households* with wealth above and below the median household in all communities (Column 3), as well as separately estimated in wealthier (Column 4) and less wealthy (Column 5) communities. Values below the median for each respective variable are labelled as 'Low'. Control means are calculated using endline data. Household controls are: age, gender, education attainment and employment status of the household head; household size; whether the household has at least one child below age 6; household wealth asset score; and whether farming is the household's main economic activity. Standard errors are clustered at the community level. Naive (unadjusted) p-values are shown in parentheses. In brackets we present p-values adjusted by family-wise error rate following Romano and Wolf (2005), using 1,000 cluster bootstrap samples. *$p < 0.10$, ** $p < 0.05$, *** $p < 0.01$.

Community- or household-level heterogeneity?

Unsurprisingly, wealthier communities are composed of a higher fraction of households with higher wealth (rather than few extremely rich individuals), and vice versa, implying that community wealth estimates may be picking up heterogeneous household-level impacts of CLTS. We thus estimate

heterogeneous impacts by household instead of community wealth. CLTS is more effective among less wealthy households compared to wealthier ones, but the estimated difference is about half as large as the community wealth impacts and not statistically significant under multiple hypothesis testing (Column 3 in Table 5).[18]

Despite the – by construction – positive correlation between individual and community wealth, significant heterogeneity remains: 31% (34%) of households living in less (more) wealthy clusters have higher (lower) wealth than the median. Splitting the sample into four cells along median community and household wealth, we further investigate CLTS impacts by household *and* community wealth. While both less *and* more wealthy households reduce OD in less wealthy communities, there is no discernible effect nor a difference between them in wealthier communities (see Table 5, Columns 4 and 5). We conclude that CLTS is more effective in less wealthy communities, regardless of the household's position in the wealth distribution. This result is in line with Ravallion and Wodon (1999), supporting the idea of geographical targeting of development interventions, which we discuss further in Section 7.

Characteristics of less and more wealthy communities

Community wealth also correlates with a number of other community characteristics, many of which have been identified as driving factors behind intervention effectiveness, particularly in the domain of public health and health-related infrastructure (Augsburg et al., 2022, Bulthuis et al., 2020, Cameron et al., 2019, Deserranno et al., 2019). We thus examine whether rural communities' (i) perceived benefits and risks of sanitation infrastructure, (ii) social cohesion and interactions, (iii) local public infrastructure and (iv) leaders' characteristics are driving our estimated wealth-specific CLTS impacts.[19]

Column 1 in Table 6 presents estimates of impact heterogeneity by communities' perceptions of sanitation benefits. We find weak evidence that CLTS impacts are high in areas where residents

[18] In Online Appendix Section E.2, we show that other household characteristics (some of which correlated with wealth), such as household composition or education of the household head, do not explain the impact of CLTS either.

[19] See Online Appendix Table E1 for an overview of systematic differences between less and more wealthy communities, and Online Appendix Section B.2 for a detailed discussion of the measurements used in this section.

perceive sanitation benefits to be low at baseline (Column 1), but only when considering naive p-values, and we do not find evidence for differential CLTS impacts between communities with high and low benefit perceptions.

Using a wide array of measurements to capture social cohesion and social interactions – community participation, trust, religious fragmentation and wealth inequality– we find slightly stronger reductions in OD in treated communities with lower baseline social capital, fragmentation and inequality, that are, however, not statistically significant (Columns 2 to 5).

Table 6
Sensitivity analysis: impact heterogeneity by communities' sanitation benefit perceptions and social interactions

Dep.variable: main respondent performs OD					
Community characteristic at baseline	Benefits	Trust	Community participation	Fragmentation	Inequality
	(1)	(2)	(3)	(4)	(5)
CLTS x Low	-0.04*	-0.04	-0.02	-0.04*	-0.05*
p-value (naive)	(0.09)	(0.11)	(0.47)	(0.07)	(0.10)
p-value (FWE corrected)	[0.50]	[0.53]	[0.94]	[0.42]	[0.51]
Difference	0.01	0.02	-0.03	0.02	0.03
p-value (naive)	(0.69)	(0.64)	(0.30)	(0.52)	(0.42)
p-value (FWE corrected)	[0.94]	[0.94]	[0.89]	[0.94]	[0.94]
Control mean (Low)	0.43	0.44	0.44	0.49	0.56
Control mean (High)	0.52	0.52	0.53	0.47	0.41
Communities	246	246	246	246	246
Observations	12,697	12,697	12,697	12,697	12,697

Notes: 'Difference' is the coefficient γ_d from estimating equation 2. Heterogeneity dimensions considered are communities' perceived sanitation benefits (Column 1), mean trust in neighbours (2), community participation (3), fragmentation (4), and wealth inequality (5). Values below the median for each respective variable are labelled as 'Low'. Control means are calculated using endline data. Household controls are: age, gender, education attainment and employment status of the household head; household size; whether the household has at least one child below age 6; household wealth asset score; and whether farming is the household's main economic activity. Standard errors are clustered at the community level. Naive (unadjusted) p-values shown in parentheses. In brackets we present p-values adjusted by family-wise error rate following Romano and Wolf (2005), using 1,000 cluster bootstrap samples. *$p<0.10$, ** $p<0.05$, *** $p<0.01$.

Our main results could also be explained by lower access to infrastructure in less wealthy communities, which we establish along the existence of local schools, hospitals and paved internal roads. For example, road infrastructure may proxy for transport costs, while health infrastructure may proxy for local hygiene knowledge and education levels. In contrast to our findings, both channels would lead us to expect higher CLTS impacts in wealthier communities. In addition, Augsburg et al. (2022) argue that poor public infrastructure may hamper the maintenance of

sanitation investments, and hence the sustainability of intervention impacts. Yet, we find no heterogeneous CLTS impacts along any public infrastructure domain (Table 7, Columns 1 to 3).

Finally, recent evidence regarding implementing interventions at scale emphasizes the importance of political leaders and implementers for their effectiveness (Cameron et al., 2019, Deserranno et al., 2019, Jack and Recalde, 2015). Particularly since leaders are the initial point of contact for the implementing WASH officials and help organize the CLTS meeting in their village, their characteristics may affect CLTS impacts. Communities with wealth below the median in our sample have less experienced and less educated leaders than those above the median, while there is no discernible difference in their political ideology (see Online Appendix Table E1). CLTS, which first seeks contact with village leaders to convince them of the benefits of sanitation, may close the knowledge gap between less and more experienced and educated leaders regarding sanitation, and hence render CLTS more effective in less wealthy communities. Yet, we find no evidence of heterogeneous CLTS impacts by leaders' tenure or education to support this hypothesis (Table 7, Columns 4 and 5).

Taken together, our results suggest that community wealth encompasses a number of distinctive community characteristics that made CLTS more effective. Yet, none of these characteristics (such as toilet coverage, implementation, measures of social cohesion, local public infrastructure or the characteristics of their leaders) could independently account for the strong differential impacts of CLTS in less wealthy communities, leaving us with one very robust predictor of CLTS effectiveness: community wealth.

Table 7
CLTS impacts on OD by community infrastructure and village leader characteristics

Dep.variable: main respondent performs OD					
Community characteristic at baseline:	Public goods			Leader traits	
	Road	Hospital	School	Experience	Education
	(1)	(2)	(3)	(4)	(5)
CLTS x Low	-0.02	-0.04**	-0.04	-0.04*	-0.03
p-value (naive)	(0.44)	(0.04)	(0.18)	(0.07)	(0.24)
p-value (FWE corrected)	[0.96]	[0.31]	[0.74]	[0.41]	[0.87]
Difference	-0.04	0.03	0.01	0.01	-0.01
p-value (naive)	(0.23)	(0.45)	(0.73)	(0.78)	(0.70)
p-value (FWE corrected)	[0.81]	[0.96]	[0.97]	[0.97]	[0.97]
Control mean (Low)	0.51	0.49	0.47	0.50	0.53
Control mean (High)	0.45	0.40	0.48	0.46	0.43
Communities	235	233	235	232	232
Observations	11,901	11,793	11,901	11,619	11,692

Notes: 'Difference' is the coefficient γ_d from estimating equation 2. Heterogeneity dimensions considered are communities' public good infrastructure (Columns 1 to 3) and leader traits (4 and 5). Values below the median of the respective community or leader characteristics are labelled as 'Low'. Control means are calculated using endline data. Household controls are: age, gender, education attainment and employment status of the household head; household size; whether the household has at least one child below age 6; household wealth asset score; and whether farming is the household's main economic activity. Standard errors are clustered at the community level. Naive (unadjusted) *p*-values shown in parentheses. In brackets we present *p*-values adjusted by family-wise error rate following Romano and Wolf (2005), using 1,000 cluster bootstrap samples. **p <0.10, ** p <0.05, *** p <0.01.*

7 Assessing external validity and the scope for wealth-based targeting of CLTS

Our finding that CLTS works in some but not in other contexts adds to the conflicting evidence on CLTS effectiveness, discussed previously. Importantly though, we identify one factor – community wealth – that can serve as guidance in which Nigerian settings CLTS can be effective in reducing open defecation.

We argue that our estimates can be used by the Government of Nigeria to target CLTS implementation, and thereby make more effective use of constrained government funds. Readily available population survey data, such as the 2013 Demographic and Household Survey (DHS), contain geographic location information and similar (if less detailed) asset lists to elicit wealth. We show in support of this argument that our findings are robust to using the less comprehensive wealth definition used in the DHS (see Online Appendix F for a detailed discussion). We use the DHS

wealth index to classify the country's regions[20] into 'low wealth' and 'high wealth' areas to obtain the targeting map shown in Figure 3. It highlights priority areas for targeting, i.e. less wealthy areas, in darker shades.[21]

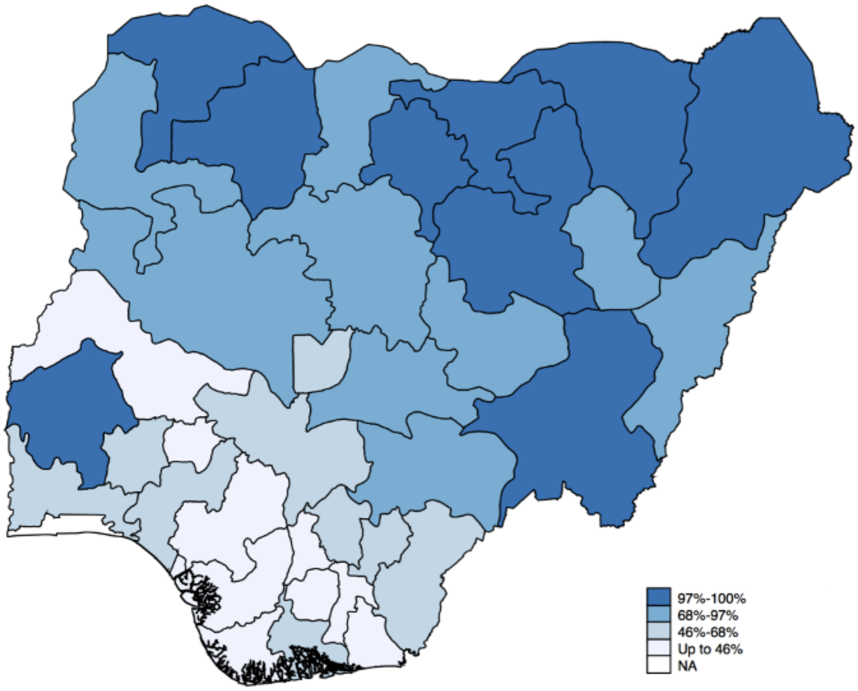

FIGURE 3
CLTS targeting in Nigeria

Notes: The map depicts the targeting map based on quartiles of the percentage of rural communities per region with wealth below the Nigerian median of community wealth. Community wealth is constructed using the DHS asset ownership list discussed in detail in Online Appendix F. Areas marked in darker shades depict regions in the lowest quartile of the Nigerian distribution of rural community wealth, i.e. those with the largest fraction of less wealthy rural communities. Based on our CLTS impact estimates by community wealth, CLTS should first prioritize these regions (where 97% or more communities have below median wealth), then those in the second quartile (where 68 to 97% of rural communities are less wealthy), and so forth. *Source:* Own calculations based on DHS Nigeria 2013.

We further conduct an exploratory analysis to get insight into whether community or area wealth could also be used as a basis for targeting CLTS implementation in other contexts.

[20] DHS is designed to be representative at the region level. Within regions, a random sample of communities is surveyed. A more detailed targeting map would require an alternative data source which covers all communities.

[21] While we conduct this analysis at a very high geographic level, the same logic can be applied with data available for lower-level geographical asset information.

In a simple cross-study analysis, we add available microdata from RCTs conducted in Indonesia and Tanzania[22] to our study data, and re-estimate impacts on this pooled sample.[23]

The exercise necessitates a consistent measure of community wealth across contexts. Since durable items underlying asset wealth indices are highly country- and context-specific (Filmer and Pritchett, 2001), so that the asset lists used in data collection vary across studies[24], we base our analysis on night light intensity, which is readily available, consistently measured globally, and has been used otherwise as a proxy for wealth or, more generally, economic activity (Michalopoulos, 2013). The CLTS impact estimates in our RCT are robust to using local night light intensity as a proxy for community wealth (see column 2, Table 5) – yet this measure does not dominate community wealth as a predictor of CLTS impacts in a horserace exercise (see Online Appendix Table G2).

We compute night light intensity at cluster level d in the baseline survey year for the pooled sample and estimate average and heterogeneous CLTS impacts on toilet ownership by night light intensity, akin to impacts presented for Nigeria in Appendix Table G3. Columns 1 and 2 in Table 8 show average impact estimates of CLTS in the pooled dataset. Country fixed effects are included in Column 2 to pick up sampling variation across RCT sites, since we cannot rely on strict exogeneity by randomization for identification when using pooled estimates. The estimated average CLTS impact is a 5 ppt increase in toilet ownership that is statistically significant and robust to the inclusion of country fixed effects.

Columns 3 to 5 present heterogeneous impact estimates for the pooled sample using three alternative functional forms to capture night light variation. First, we split geographic units according

[22]The datasets can be found in: Water and Sanitation Program (2008) for Indonesia and Briceno et al. (2012) for Tanzania.

[23]We acknowledge that each of these study contexts will be additionally characterized by context or location-specific factors (and measurement error), emphasized in Wang et al. (2006) and Meager (2019), and that community wealth may not be the unique source of heterogeneous impacts. A more detailed analysis would require access to the exact cluster locations in a larger number of studies than currently available to identify cross-study variation separately from genuine observational differences across households as, for example, proposed by Meager (2019). Unfortunately, microdata from the CLTS RCT in Mali are not publicly available, and data from the RCT in Bangladesh do not provide cluster level identifiers (Guiteras et al., 2017). We use these data in a coarser analysis, described in Online Appendix Section G.2. We estimate heterogeneous CLTS impacts by night light intensity separately in all five RCTs, using the lowest available geographical observation unit to map in night light intensity. We consistently find that CLTS is on average more effective in less wealthy settings, i.e. those with lower night light intensity.

[24]The Indonesia study, for example, provides only a monetary value of all assets considered, and in Tanzania the list of asset information is quite limited.

Table 8
Pooled CLTS impacts by night light

Dep. variable: toilet ownership	All		By area nightlight intensity (NL)		
			Zero vs. pos. nightlight	Below vs. above Nigerian median	NL tertiles
	(1)	(2)	(3)	(4)	(5)
Panel A - Pooled average impact					
CLTS	0.05**	0.05**			
	(0.01)	(0.01)			
Panel B - Heterogeneity by communities' nightlight intensity					
CLTS in zero/below median/1st tertile NL			0.09***	0.12***	0.09***
			(0.01)	(0.00)	(0.01)
CLTS in 2nd tertile NL					0.07**
					(0.04)
CLTS in positive/above median/3rd tertile NL			0.03	-0.01	-0.01
			(0.25)	(0.62)	(0.69)
Difference (High-Low)			-0.06	-0.13***	-0.10**
			(0.14)	(0.00)	(0.03)
Difference (Middle-Low)					-0.02
					(0.71)
Country FE	No	Yes	Yes	Yes	Yes
Communities	580	580	580	580	580
Observations	7,843	7,843	7,843	7,843	7,843

Notes: Pooled regression results using the Indonesian, Nigerian and Tanzanian samples. All specifications control for gender, age and age squared of the household head, as well as whether farming is the main economic activity of the household. District fixed effects are also included and errors are clustered at the level of the randomization unit. p-values are shown in parentheses. **p <0.10, ** p <0.05, *** p <0.01.*

to whether they display zero or positive night light intensity to reflect the strong right skew of its distribution (Appendix Figure B1). Second, we use the results from our Nigerian RCT as reference and define the Nigerian night light median at the community level as a split point as we found no CLTS impact estimates beyond the median level of wealth in our RCT (Figure 1 and Table 3). Third, we estimate heterogeneous impacts by night light intensity using a more flexible split into tertiles. We replicate our finding that CLTS impacts vary by communities' wealth status, using this pooled sample and night light intensity as a proxy. Impact estimates are substantially larger in areas with low night light intensity, of magnitude 9 ppts in areas with zero night light, i.e. the lowest tertile.[25] These results get even stronger when we split areas along the Nigerian night light median (Column 4): in areas of low night light intensity CLTS increases toilet ownership by 12 percentage points (significant at the 1% confidence level). Similarly, we never find statistically significant CLTS impact estimates in areas of high night light intensity in any specification. Furthermore, impacts are declining across tertiles of increasing night light (Column 5), similar to the results in our RCT presented previously (Figure 1). We test whether the difference between the estimated coefficients in 'low wealth' and 'high wealth' areas differ from zero and reject the hypothesis in all specifications but one. The exception is the specification in Column 3 where we split the sample into zero and positive night light areas. It is likely that this split is too coarse, since it puts areas with very low but positive night lights into the 'high' category. [26]

We supplement this evidence by plotting point estimates of impacts on toilet ownership and open defecation observed in our study, Tanzania and Indonesia (Briceno et al., 2017, Cameron et al., 2019), as well as two further RCT evaluations, in Bangladesh and Mali (Guiteras et al., 2015, Pickering et al., 2015) by their corresponding satellite night light intensity. The exercise yields an inverse relationship between community wealth and CLTS impacts (see Online Appendix, Figure G1).

In summary, the pooled cross-context estimates closely replicate our CLTS impact estimates from the Nigerian setting, and suggest that our findings may also be useful for the targeting of

[25] 31% of the observations in the pooled sample are located in areas with zero night light intensity.

[26] We show results for a similar analysis using toilet coverage as a measure of heterogeneity and we find no significant differences. See Table G4 in the Appendix.

CLTS beyond the Nigerian context.

8 Conclusion

The design of effective policies to address the urgent sanitation concerns in the developing world requires a nuanced understanding of households' investment choices and drivers of behavioral change. In this paper we provide evidence on the effectiveness of Community-Led Total Sanitation (CLTS), a participatory information intervention without financial components, as implemented at scale in a collaboration between NGOs and the Nigerian government.

Our study uses a large cluster randomized experiment in Nigeria for which we collected data up to three years after treatment. Implementation of CLTS in this context was conducted at scale, i.e. by WASH civil servants trained by local NGOs. We show that the intervention had strong heterogeneous impacts by community wealth, with significant and lasting effects on open defecation habits in less wealthy communities, reducing OD rates by 9 percentage points from a baseline level of 75%. We find no effect of CLTS in wealthier communities. The OD reduction in less wealthy communities is achieved mainly through increased toilet ownership (+8 ppts from a baseline level of 24%). This result, which is robust across alternative measures of community socio-economic status, is not driven by baseline differences in toilet coverage, and can be replicated across other settings, which we show by pooling data from our study and a limited set of RCTs of similar interventions.

We provide an example of how our results could be used to develop a potentially more effective targeting strategy for CLTS in Nigeria as well as other contexts.

Our results have two further implications. First, they provide an additional reason why scale-up of interventions is not trivial (Banerjee et al., 2017, Bold et al., 2018, Deaton and Cartwright, 2018, Ravallion, 2012). Discussions on why interventions may not scale-up successfully in a national roll-out have focused on general equilibrium and spillover effects, and recently on aspects of implementation and delivery. We show, in line with the literature on geographical poverty traps, that community-specific, heterogeneous treatment impacts are an additional impediment to successful scale-up in terms of effectiveness of interventions.

Second, we show that interventions relying on information and collective action mechanisms

can have substantial impacts on households' health investments and behavior, specifically relating to sanitation. Yet, there is an important caveat for policymakers working towards meeting the sanitation-related sustainable development goals. CLTS achieves convergence between asset-poor and asset-rich communities in terms of OD and toilet coverage in our study, and thus levels the playing field. However, it is not a silver bullet that closes the large sanitation gap towards achieving open-defecation-*free* status in less wealthy communities as a standalone intervention. Hence, more research on alternative or supplementary interventions to close the sanitation gap in low-income countries is needed. These may seek to magnify CLTS impacts achieved through geographical targeting by complementing with individually targeted interventions (Elbers et al., 2007). We know from the literature that the alleviation of liquidity constraints (through financial incentives, loans or subsidies) is important (see, for example, Andres et al. (2020), Ben Yishay et al. (2017), Guiteras et al. (2015), Patil et al. (2014)), as are implementation design choices, such as more intensive follow-up (Augsburg et al., 2022, Venkataramanan et al., 2018).

In addition, there should be a focus on alternative approaches in wealthier communities where CLTS is ineffective, for example, via infrastructure investment and supply-side interventions.

References

Abramovsky, L., Augsburg, B., and Oteiza, F. (2015). *Baseline report: Sustainable Total Sanitation - Nigeria*. Institute for Fiscal Studies, London.

Adukia, A. (2017). Sanitation and education. *American Economic Journal: Applied Economics*, 9(2):23–59.

Alesina, A., Baqir, R., and Easterly, W. (1999). Public goods and ethnic divisions. *Quarterly Journal of Economics*, 114(4):1243–1284.

Alesina, A. and La Ferrara, E. (2002). Who trusts others? *Journal of Public Economics*, 85(2):207–234.

Alsan, M. and Goldin, C. (2019). Watersheds in Child Mortality: The Role of Effective Water and Sewerage Infrastructure, 1880-1920. *Journal of Political Economy*, 127(2):586–638.

Alzúa, M. L., Djebbari, H., and Pickering, A. J. (2020). A community-based program promotes sanitation. *Economic Development and Cultural Change*, 68(2):357–390.

Andres, L. A., Deb, S., Joseph, G., Larenas, M. I., and Grabinsky Zabludovsky, J. (2020). A multiple-arm, cluster-randomized impact evaluation of the Clean India (Swachh Bharat) Mission program in rural Punjab, India. World Bank Policy Research Working Paper 9249.

Angrist, J. D. and Imbens, G. W. (1995). Two-stage least squares estimation of average causal effects in models with variable treatment intensity. *Journal of the American Statistical Association*, 90(430):431–442.

Augsburg, B., Bancalari, A., Durrani, Z., Vaidyanathan, M., and White, Z. (2022). When nature calls back: Sustaining behavioral change in rural Pakistan. *Journal of Development Economics*, 158:102933.

Augsburg, B., Caeyers, B., Giunti, S., Malde, B., and Smets., S. (2021a). Labelled loans and human capital investments. IFS Working Paper W21/09.

Augsburg, B., Malde, B., Olorenshaw, H., and Wahhaj, Z. (2021b). To invest or not to invest in sanitation: the role of intra-household gender differences in perceptions and bargaining power. Technical report, IFS Working Paper.

Augsburg, B. and Rodriguez-Lesmes, P. A. (2018). Sanitation and child health in India. *World Development*, 107:22–39.

Baird, S., Hicks, J. H., Kremer, M., and Miguel, E. (2016). Worms at work: Long-run impacts of a child health investment. *The Quarterly Journal of Economics*, 131(4):1637–1680.

Banerjee, A., Banerji, R., Berry, J., Duflo, E., Kannan, H., Mukherji, S., Shotland, M., and Walton, M. (2017). From proof of concept to scalable policies: Challenges and solutions, with an application. *Journal of Economic Perspectives*, 31(4):73–102.

Ben Yishay, A., Fraker, A., Guiteras, R., Palloni, G., Shah, N. B., Shirrell, S., and Wang, P. (2017). Microcredit and willingness to pay for environmental quality: Evidence from a randomized-controlled trial of finance for sanitation in rural Cambodia. *Journal of Environmental Economics and Management*, 86:121–140.

Bold, T., Kimenyi, M., Mwabu, G., Ng'ang'a, A., and Sandefur, J. (2018). Experimental evidence on scaling up education reforms in Kenya. *Journal of Public Economics*, 168:1–20.

Briceno, B., Coville, A., Gertler, P., and Martinez, S. (2017). Are there synergies from combining hygiene and sanitation promotion campaigns: Evidence from a large-scale cluster-randomized trial in rural Tanzania. *PLoS One*, 12(11):e0186228.

Briceno, B., Coville, A., and Martinez, S. (2012). Impact Evaluation of Scaling-up Handwashing and Rural Sanitation Behavior Projects in Tanzania 2012, Endline Survey (SHRSBIE-EL). Ref. TZA_2012_SHRSBIE-EL_v01_M_v01_A_PUF.

Brown, J., Albert, J., and Whittington, D. (2019). Community-led total sanitation moves the needle on ending open defecation in Zambia. *American Journal of Tropical Medicine and Hygiene*, 100(4):767–769.

Bulthuis, S. E., Kok, M. C., Raven, J., and Dieleman, M. A. (2020). Factors influencing the scale-up of public health interventions in low- and middle-income countries: a qualitative systematic literature review. *Health Policy and Planning*, 35(2):219–234.

Cameron, L., Olivia, S., and Shah, M. (2019). Scaling up sanitation: Evidence from an RCT in Indonesia. *Journal of Development Economics*, 138:1–16.

Cohen, J. and Dupas, P. (2010). Free distribution or cost-sharing? Evidence from a randomized malaria prevention experiment. *Quarterly Journal of Economics*, 125(1):1–45.

Crocker, J., Saywell, D., and Bartram, J. (2017). Sustainability of community-led total sanitation outcomes: Evidence from Ethiopia and Ghana. *International Journal of Hygiene and Environmental Health*, 220(3):551–557.

Deaton, A. and Cartwright, N. (2018). Understanding and misunderstanding randomized controlled trials. *Social Science & Medicine*, 210:2–21.

Deserranno, E., Stryjan, M., and Sulaiman, M. (2019). Leader selection and service delivery in community groups: Experimental evidence from uganda. *American Economic Journal: Applied Economics*, 11(4):240–67.

Dupas, P. (2011). Health behavior in developing countries. *Annual Review of Economics*, 3(1):425–449.

Easterly, W. and Levine, R. (1997). Africa's growth tragedy: Policies and ethnic divisions. *Quarterly Journal of Economics*, 112(4):1203–1250.

Elbers, C., Fuji, T., Lamjouw, P., Ozler, B., and Yin, W. (2007). Poverty alleviation through geographic targeting. *Journal of Development Economics*, 83:198–213.

Filmer, D. and Pritchett, L. (2001). Estimating wealth effects without expenditure data–or tears: An application to educational enrollments in states of India. *Demography*, 38(1):115–132.

Guiteras, R., Levinsohn, J., and Mobarak, A. M. (2015). Sanitation subsidies. Encouraging sanitation investment in the developing world: a cluster-randomized trial. *Science*, 348(6237):903–906.

Guiteras, R., Levinsohn, J., and Mobarak, A. M. (2017). Encouraging Sanitation Investment in the Developing World: A Cluster-Randomized Trial - Dataset. *Harvard Dataverse*.

Gupta, A., Khalid, N., Deshpande, D., Hathi, P., Kapur, A., Srivastav, N., Vyas, S., Spears, D., and Coffey, D. (2020). Revisiting open defecation evidence from a panel survey in rural North India, 2014–18. *Economic & Political Weekly*, 55(21):55–63.

Henderson, J. V., Storeygard, A., and Weil, D. N. (2012). Measuring economic growth from outer space. *American Economic Review*, 102(2):994–1028.

Imbens, G. W. and Angrist, J. D. (1994). Identification and estimation of local average treatment effects. *Econometrica*, 62(2):467–475.

Jack, B. K. and Recalde, M. P. (2015). Leadership and the voluntary provision of public goods: Field evidence from Bolivia. *Journal of Public Economics*, 122:80–93.

Jalan, J. and Ravallion, M. (2002). Geographic poverty traps? a micro model of consumption growth in rural China. *Journal of Applied Econometrics*, 17(4):329–346.

Kar, K. (2003). Subsidy or self-respect? Participatory total community sanitation in Bangladesh. IDS Working Paper.

Kar, K. and Chambers, R. (2008). *Handbook on Community-Led Total Sanitation*, volume 44. Institute of Development Studies, Brighton.

Khanna, T. and Das, M. (2016). Why gender matters in the solution towards safe sanitation? Reflections from rural India. *Global Public Health*, 11(10):1185–1201.

Mauro, P. (1995). Corruption and growth. *The Quarterly Journal of Economics*, 110(3):681–712.

McKenzie, D. (2012). Beyond baseline and follow-up: The case for more T in experiments. *Journal of Development Economics*, 99(2):210–221.

McKenzie, D. J. (2005). Measuring inequality with asset indicators. *Journal of Population Economics*, 18(2):229–260.

Meager, R. (2019). Understanding the average impact of microcredit expansions: A Bayesian hierarchical analysis of seven randomized experiments. *American Economic Journal: Applied Economics*, 11(1):57–91.

Michalopoulos, S. E. P. (2013). Pre-colonial ethnic institutions and contemporary African development. *Econometrica*, 81(1):113–152.

Miller, G. and Mobarak, A. M. (2013). *Gender Differences in Preferences, Intra-Household Externalities, and Low Demand for Improved Cookstoves.* NBER, Cambridge, MA.

Muralidharan, K. and Niehaus, P. (2017). Experimentation at scale. *Journal of Economic Perspectives*, 31(4):103–24.

Orgill-Meyer, J. and Pattanayak, S. (2020). Improved Sanitation Increases Long-Term Cognitive Test Scores. *World Development*, 132:104975.

Orgill-Meyer, J., Pattanayak, S. K., Chindarkar, N., Dickinson, K. L., Panda, U., Rai, S., Sahoo, B., Singha, A., and Jeuland, M. (2019). Long-term impact of a community-led sanitation campaign in India, 2005–2016. *Bulletin of the World Health Organization*, 97:523–533A.

Patil, S. R., Arnold, B. F., Salvatore, A. L., Briceño, B., Ganguly, S., Colford, J. M., and Gertler, P. J. (2014). The effect of India's Total Sanitation Campaign on defecation behaviors and child health in Rural Madhya Pradesh: A cluster randomized controlled trial. *PLOS Medicine*, 11(8):e1001709.

Pattanayak, S. K., Yang, J.-C., Dickinson, K. L., Poulos, C., Patil, S. R., Mallick, R. K., Blitstein, J. L., and Praaharaj, P. (2009). Shame or subsidy revisited: social mobilization for sanitation in Orissa, India. *Bulletin of the World Health Organization*, 87:580–587.

Pickering, A. J., Djebbari, H., Lopez, C., Coulibaly, M., and Alzua, M. L. (2015). Effect of a community-led sanitation intervention on child diarrhoea and child growth in rural Mali: A cluster-randomised controlled trial. *The Lancet Global Health*, 3(11):e701–e711.

Prüss-Ustün, A., Bartram, J., Clasen, T., Colford, J. M., Cumming, O., Curtis, V., Bonjour, S., Dangour, A. D., Fewtrell, L., DeFrance, J., Fewtrell, L., Freeman, M. C., Gordon, B., Hunter, P. R., Johnston, R. B., Mathers, C., Mäusezahl, D., Medlicott, K., Neira, M., Stocks, M., Wolf, J., and Cairncross, S. (2014). Burden of disease from inadequate water, sanitation and hygiene in low- and middle-income settings: A retrospective analysis of data from 145 countries. *Trop Med Int Health (in submission)*, 19(8):894–905.

Radin, M., Jeuland, M., Wang, H., and Whittington, D. (2020). Benefit–cost analysis of community-led total sanitation: Incorporating results from recent evaluations. *Journal of Benefit-Cost Analysis*, 11(3):380–417.

Ravallion, M. (2012). Fighting poverty one experiment at a time: Poor economics: A radical rethinking of the way to fight global poverty: Review essay. *Journal of Economic Literature*, 50(1):103–114.

Ravallion, M. and Wodon, Q. (1999). Poor areas, or only poor people? *Journal of Regional Science*, 39(4):689–711.

Romano, J. P. and Wolf, M. (2005). Stepwise multiple testing as formalized data snooping. *Econometrica*, 73(4):1237–1282.

Sahoo, K., Hulland, K., Caruso, B., Swain, R., Freeman, M., Panigrahi, P., and Dreibelbis, R. (2015). Sanitation-related psychosocial stress: A grounded theory study of women across the life-course in Odisha, India. *Social Science & Medicine*, 139:80–89.

Spears, D. and Lamba, S. (2016). Effects of early-life exposure to sanitation on childhood cognitive skills: Evidence from india's total sanitation campaign. *Journal of Human Resources*, 51(2):298–327.

Venkataramanan, V., Crocker, J., Karon, A., and Bartram, J. (2018). Community-led total sanitation: A mixed-methods systematic review of evidence and its quality. *Environmental Health Perspective*, 126(2):026001.

Wang, S., Moss, J., and Hiller, J. (2006). Applicability and transferability of interventions in evidence-based public health. *Health Promotion International*, 21(1):76–83.

Water and Sanitation Program (2008). Indonesia WSP Global Scaling Up Rural Sanitation Access (TSSM) Impact Evaluation, Baseline and Endline Surveys 2009-2011. Ref. IDN_2009_2011_WSP-IE_v01_M_v01_A_PUF.

WHO/UNICEF (2021). *Progress on Drinking Water, Sanitation and Hygiene: 2000-2020. Five years into the SDGs*. Geneva.

World Bank (2019). *World Development Report 2019: The Changing Nature of Work*. Washington, DC: World Bank. doi:10.1596/978-1-4648-1328-3. License: Creative Commons Attribution CC BY 3.0 IGO.

Zuin, V., Delaire, C., Peletz, R., Cock-Esteb, A., Khush, R., and Albert, J. (2019). Policy diffusion in the rural sanitation sector: Lessons from Community-Led Total Sanitation (CLTS). *World Development*, 124:104643.

ONLINE APPENDICES

Community Matters:

Heterogeneous Impacts of a Sanitation Intervention

Laura Abramovsky, Britta Augsburg, Melanie Lührmann, Francisco Oteiza, Juan Pablo Rud

A Study design

We conducted our study as an RCT in four LGAs without recent experience of CLTS or CLTS-like interventions in Enugu and five LGAs in Ekiti. Their locations are indicated in Figure A1.[2]

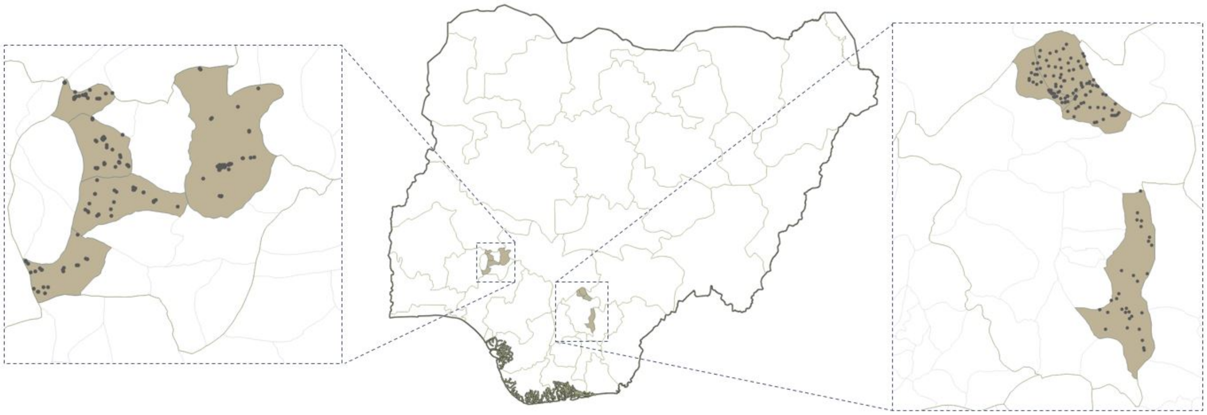

Figure A1
Map of the study area in Ekiti and Enugu

Note: Study clusters in the nine LGAs (shaded) in Ekiti (left) and Enugu (right). Indicated dots are locations of study communities.

246 clusters were randomized with equal probability into either receiving CLTS (treatment) or not receiving it during the course of the study (control). Randomization was stratified by LGA. The distribution of treatment and control clusters is presented in Table A1.

The timing of the intervention and our data collection is summarised in Figure A2.

[2] Study LGAs in Enugu are Igbo Eze North, Igbo Eze South, Nkanu East and Udenu. In Ekiti, Ido Osi, Ikole, Moba, Irepodun Ifelodun and Ekiti South West are part of the study.

Table A1

Number of clusters per study arm and state

	Control		CLTS		Total	
	Freq.	%	Freq.	%	Freq.	%
Ekiti	63	51.6	66	52.8	129	52.2
Enugu	59	48.4	59	47.2	118	47.8
Total	122	100.0	125	100.0	247	100.0

Notes: Numbers and percentages of communities allocated to the two experimental arms (control and CLTS) are presented. Of the 247 identified communities, one was subsequently dropped since data collection in any post-treatment wave was not possible due to civil unrest.

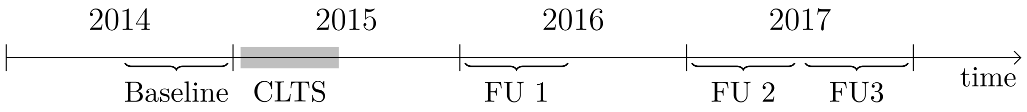

Figure A2

Timeline of intervention implementation and data collection

Notes: 'CLTS' stands for Community-Led Total Sanitation, 'FU' for follow-up survey. The grey bar indicates the time when CLTS was implemented in treatment communities.

B Variable definitions and measurements

In this section, we provide details on a series of measurements used to construct household- and community-level characteristics. These are based on our household surveys and other auxiliary datasets.

B.1 Household characteristics

Asset wealth

Household survey measures of annual household income had relatively low response rates: 27% of the households interviewed reported no income at all or refused to answer. A higher response rate was achieved in a list of questions regarding the ownership of consumer durables. We applied a principal component analysis to this list, and constructed an index of wealth based on the first principal component, following Filmer and Pritchett (2001). Such wealth indices are widely used as a proxy for household long-term wealth, for example, in the USAID Demographic and Health Surveys (DHS) run over 90 countries, or as a targeting tool for the PROGRESA conditional cash-

transfer programme (McKenzie, 2005). Table B1 lists the asset items elicited in our household survey, and shows their factor loadings.

Table B1
Asset items used in the wealth index

Ownership of the following durable assets: (1=Yes, 0=No)	
Asset	Factor loading
Motorcycle/scooter/tricycle	0.1302
Furniture: chairs	0.1561
Furniture: tables	0.1823
Furniture: beds	0.1075
Refrigerator	0.2998
Washing machine	0.1826
Microwave oven	0.1914
Gas cooker	0.2507
Plasma/flat screen TV	0.2173
Other TV	0.2867
Satellite dish (monthly subscription)	0.2272
Other satellite dish (DSTV, etc.)	0.2391
Radio/CD/DVD player	0.2241
Smart phones	0.1265
Other phones	0.0886
Computer	0.2195
Air conditioner	0.1061
Power generator	0.2777
Sewing machine	0.1323
Electric iron	0.3172
Pressure cooker	0.1557
Electric fans	0.3162

Notes: Questions were coded equal to 1 if the household reported owning at least one of each of the items listed in each category. The wealth index was then constructed using the first component of the principal component analysis. Households with missing data for at least one of the categories were excluded.

B.2 Community characteristics

A community is on average composed of 1.7 villages or quarters, comprising 220 households.

Community wealth

Community wealth is estimated as the median household's wealth score. Our household survey randomly interviewed 20 households per community, so we chose the median to limit possible distortions due to outliers (i.e. households with extremely high or low wealth). Our main results discretize community wealth along the median into less and more wealthy ones.

Night light index

As an alternative measure of communities' socio-economic status, we use the average night light index recorded in 2013, before the intervention began. Based on household GPS coordinates, we calculate the geographical centroid of each community, and define a 5km radius around the centroid. We used night time light data made available by the US National Oceanographic and Atmospheric Administration (NOAA). The observations on which the data are assembled are made by the Operational Linescan System (OLS) flown on the Defense Meteorological Satellite Program (DMSP) satellites.

The intensity of night time lighting has been shown to be correlated with economic growth, and was proposed as a tool for inferring growth rates at the sub-national level by Henderson et al. (2012). Moreover, Michalopoulos (2013) shows that night time light intensity is highly correlated with GDP per capita and urbanization across African countries.

Figure B1 shows the distribution of night light intensity across communities in our Nigerian study sample (and in the pooled cross-country sample). Its skewness is one reason for the discrete measures we use in our preferred specification (and for the sensitivity analysis using the continuous measures).

Toilet coverage

Toilet coverage is the share of households in the community that owned toilets at baseline. It is positively, but not very strongly correlated with wealth ($\rho = 0.5459$) and night light ($\rho = 0.2211$).

Sanitation risk and benefit perceptions

We construct an index of perceived benefits of sanitation access which is based on eight questions (listed in Table B2), each asked using a five-point Likert scale, eliciting perceptions in terms of health, well-being, productivity, time savings, pride, social status and personal safety. We also elicited (mis)perceptions of risk associated with toilet ownership, such as the belief that toilets spread infections and cause sickness.

Religious fragmentation

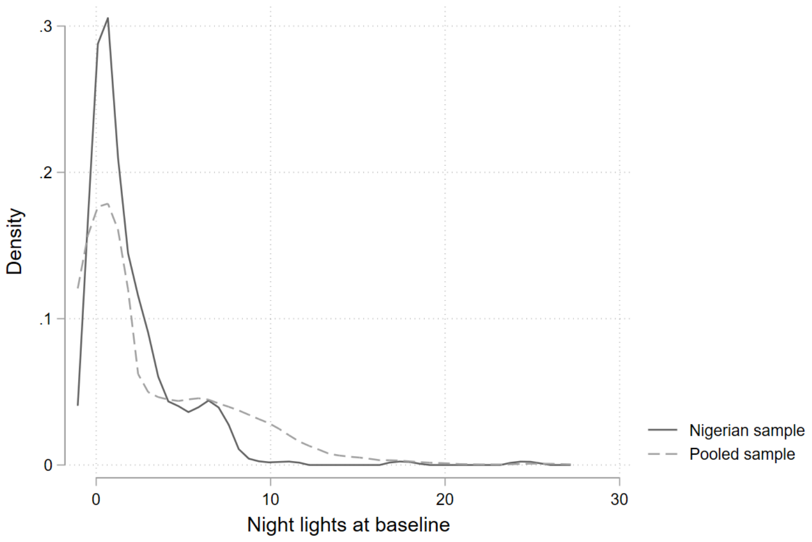

Figure B1
Distribution of night light at baseline, Nigerian and pooled samples

Note: Kernel density estimates of the distribution of night light for our Nigerian study sample and the pooled study sample comprising the study samples from Indonesia, Nigeria and Tanzania.

Religious fragmentation denotes the probability that two randomly selected households in our sample are of different religions. We focus on religious rather than ethnic fragmentation, as our study sample is extremely homogeneous along ethnic lines but exhibits considerable religious diversity.[3]

Trust

In a seminal paper, Alesina and La Ferrara (2002) define trust based on a question from the US General Social Survey, asking respondents whether they thought that 'most people can be trusted'. Since we want to uncover trust *within* the community rather than a concept of social trust, we instead rely on the following question: 'Generally speaking, would you say that you trust the people in your neighborhood a lot, only a little, or not at all?'. Responses were coded with 2 (a lot), 1 (a little) or 0 (not at all). We use the mean value of household responses within a community as the community-level aggregate. We note that while the question was extensively piloted to ensure suitability to the local context, no further validation exercises were conducted.

[3] Similarly defined indices, first used in Mauro (1995), are frequently used to study ethnic diversity and its impact on economic growth (Easterly and Levine, 1997) or public goods provision (Alesina et al., 1999).

Table B2

Questions used to construct sanitation benefit and risk indices

Survey question	Indices of Benefit	Indices of Risk
Imagine your neighbour never had a toilet/latrine. In the last month, he/she completed construction of a toilet/latrine as shown in this picture. Do you think this neighbor's family will...		
...be happier...	X	
...be healthier...	X	
...be more productive...	X	
...be less embarrassed when family and friends come to visit...	X	
...feel proud...	X	
...have a higher status in society...	X	
...feel that women in the family will be safer...	X	
...save time...	X	
...get sick more easily...		X
...see the women getting infections due to pit latrine heat...		X
...because of this toilet/latrine?		

Notes: All questions use a five point Likert scale, ranging from 'strongly agree' to 'strongly disagree'. For the purposes of constructing these indices, these responses were standardized before conducting the principal component analysis. Households with missing data for at least one of the categories were excluded.

Community participation

An index of household level community participation was constructed by principal component analysis using questions related to participation in community life, social contact and provision of services to the community (see Table B3). It is similar to the one used by Cameron et al. (2019), who study CLTS impacts in the province of East Java, Indonesia. They refer to their index as 'Social Capital'. Table B3 shows each item's factor loading. We aggregate household responses to a community-level measure by taking the community-specific mean.

Wealth inequality

Wealth inequality is constructed by dividing the *within-community* standard deviation of household wealth by the standard deviation of household wealth over the whole sample. McKenzie (2005) shows that, in the absence of reliable information on household income or consumption, this measure of wealth inequality is an informative proxy for inequality in living standards.

Communities' public infrastructure

Local school or hospital infrastructure and an indicator for whether the community has paved

Table B3

Questions used to construct the community participation index

Survey question	Factor loading
How many times in the past 12 months have you...	
...donated blood?	0.0573
...worked on a community project?	0.3129
...attended any public meeting in which there was discussion of town or school affairs?	0.2939
...attended a political meeting or rally?	0.2387
...attended any club or organizational meeting (not including meetings for work)?	0.3204
...had friends over to your home?	0.3662
...been in the home of/invited someone of a different race?	0.3166
...been in the home of/invited someone of a different neighborhood?	0.3573
...been in the home of/invited someone you consider to be a community leader?	0.3699
...volunteered?	0.3185
...served as an official or served on a committee of a club or community association?	0.2259
Not including weddings and funerals, how often do you attend religious services?	0.0382

Notes: These were multiple choice questions in which the (pre-specified) answers ranged from 'Never did this' to 'More than once a week'. For the purposes of constructing the social capital index, these responses were standardized, before conducting the principal component analysis. Households with missing data for at least one of the categories were excluded.

internal roads serve as further measures of local development status, elicited from community leaders via a community-level questionnaire conducted at baseline.

Characteristics of village leaders

CLTS is triggered after an initial meeting with the village leader who helps organize the community meeting and aims to spur collective action on towards open-defecation-free status of the community. Hence, village leaders may influence their community's characteristics. As a community may comprise several villages, these characteristics reflect the leaders' mean age, experience (years in leadership), education attainment and political ideology, measured at baseline.

C Attrition

In this section, we provide summary statistics of attrition of households in our sample across the three follow-up surveys, in treatment and control, and by community wealth (Table C1). Our study showed remarkably low attrition rates: 2.56% in FU1, 9.23% in FU2 and 12.22% in FU3; these are very similar across treatment arms. We find slightly lower attrition rates in less wealthy communities than in wealthier ones.

Table C1

Attrition rates by survey wave, treatment status and community wealth

Failed interviews as % of total attempted	Survey wave		
	FU1	FU2	FU3
Whole sample	2.56	9.23	12.22
By treatment status:			
CLTS	2.59	9.50	12.96
Control	2.53	8.97	11.48
By community level wealth:			
Wealthier communities	3.30	10.16	14.63
Less wealthy communities	1.75	8.22	9.64

Notes: Attrition rates are defined as a share of the total attempted interviews in each survey wave. FU 1, 2 and 3 refer to follow-up survey waves at 8, 24 and 32 months after baseline.

In Table C2 we formally test whether treatment status can predict attrition conditional on baseline characteristics. Columns 1, 4 and 7 show the results of a regression of attrition on the treatment indicator and district fixed effects, for waves FU1, FU2 and FU3 respectively. Assignment to CLTS does not predict attrition, and this result is robust to the inclusion of household-level controls (Columns 2, 5 and 8). Hence, we find no evidence of selective attrition that would challenge the successful randomization demonstrated in Table 1. Attrition is balanced not only across treatment groups, but also by community wealth in FU1 and FU2. Only in FU3 do we find slightly higher attrition rates (4ppts) among wealthier communities than less wealthy ones (p-value=0.00). Yet, recall that even three years after the baseline survey, mean attrition is very low at around 12%.

Table C2
Tests for non-random attrition

Survey wave:	FU1			FU2			FU3		
	(1)	(2)	(3)	(4)	(5)	(6)	(7)	(8)	(9)
Treatment status	0.00	-0.00	0.00	0.01	-0.01	-0.01	0.01	0.00	0.01
	(0.90)	(0.96)	(0.90)	(0.53)	(0.27)	(0.30)	(0.12)	(0.71)	(0.49)
Less wealthy communities			-0.01			-0.01			-0.04***
			(0.14)			(0.52)			(0.00)
Household controls	No	Yes	Yes	No	Yes	Yes	No	Yes	Yes
Observations	4,647	4,505	4,505	4,647	4,505	4,505	4,722	4,546	4,546

Notes: Estimation results for regressions using equation 1 of attrition, by wave, on treatment status, household and community characteristics. FU 1, 2 and 3 refer to follow-up survey waves at 8, 24 and 32 months after baseline. Standard errors are clustered at the community level. p-values are shown in parentheses. **p <0.10, ** p <0.05, *** p <0.01.*

D Sensitivity analysis: measures of sanitation behavior

In the main text, we showed impact estimates on open defecation based on the behavior of the main respondent. We also asked about the defecation place of every household member. We aggregate it into an indicator of whether at least one household member above the age of 4 performs OD, and present sensitivity analysis of CLTS impacts in Table D1. This measure was not elicited in the first follow-up survey; hence we report only the pooled estimates and those for FU2 and FU3. The results are qualitatively and quantitatively very similar.

Table D1
Average CLTS impacts on OD performed by any household member

Dependent variable: OD by any member	DiD		ANCOVA
	(1)	(2)	(3)
Panel A - Pooled impacts			
CLTS (γ)	-0.03	-0.03	-0.03*
	(0.25)	(0.16)	(0.06)
Panel B - Impacts over time			
CLTS x FU 2	-0.04	-0.04	-0.04*
	(0.18)	(0.11)	(0.06)
CLTS x FU 3	-0.03	-0.03	-0.03
	(0.41)	(0.30)	(0.14)
Household controls	No	Yes	Yes
Control mean	0.49	0.49	0.49
Communities	246	246	246
Observations	8,786	8,518	8,518

Notes: Control means are calculated using endline data. Panel A reports estimates using data pooled across all three post-intervention survey waves, while Panel B shows estimates separately for the follow-up survey waves conducted 24 (FU2) and 32 months (FU3) after baseline. The estimates, obtained using equation 1, provide sensitivity analysis for those in Table 2, using OD by *any* household member above the age of 4 as alternative OD measure. Household controls are: age, gender, education attainment and employment status of the household head; household size, whether the household has at least one child below age 6, household wealth asset score, and whether farming is the household's main economic activity. Standard errors are clustered at the community level. p-values shown in parentheses. *$p < 0.10$, ** $p < 0.05$, *** $p < 0.01$.*

E Sensitivity analysis: heterogeneous impacts

In this section, we present additional sensitivity analysis that underlines the robustness of the estimates presented in the main paper. Specifically, we (i) document differences in community characteristics by wealth, (ii) consider whether impacts may be driven by additional individual-level heterogeneous CLTS impacts, and (iii) demonstrate functional form robustness by re-estimating heterogeneous impacts for continuous measures of communities' asset wealth.

E.1 Differences between less and more wealthy communities

In addition to arguing that CLTS will be more effective in low SES communities, the pioneers of the approach posit that successful implementation of CLTS is more likely in rural communities that

are small, culturally and socially homogeneous, and have a high prevalence of OD. Cameron et al. (2019) emphasize communities' social capital as a key facilitator for CLTS impact in the context of Indonesia. Since these community characteristics may all correlate with community wealth, we consider whether some of these dimensions drive heterogeneous CLTS impacts.

In Table E1, we examine whether less and more wealthy communities differ at baseline along four dimensions – their perceived benefits and risks of sanitation access, social interactions, access to public infrastructure and village leader characteristics.[4] Residents of wealthier communities have similar pre-intervention perceptions that access to sanitation yields benefits – along the aspects of health, well-being, productivity, time savings, pride, social status and personal safety. Less wealthy communities exhibit higher levels of social cohesion (as measured by community-level trust and religious fragmentation) and lower levels of asset wealth inequality than wealthier ones. We also find significant differences in access to public infrastructure, including having a local school, a hospital and paved internal roads. Finally, less wealthy communities have less experienced and less educated leaders.

Common (mis)perceptions of sanitation risk, community participation and a village leader's political connectedness appear uncorrelated with community wealth.[5]

E.2 Household-level heterogeneity in CLTS impacts

In addition to heterogeneous impacts by household wealth which we investigated in Table 5, we used (completed primary) education of the household head as a proxy for household wealth, and find no evidence of heterogeneous impacts along this dimension (see Column 1 in Table E2).

Finally, it is often posited that women and households with children may exhibit a higher willingness to invest in health and sanitation technologies.[6] In columns 2 and 3 of Table E2, we

[4] Detailed definitions of these measures are in Online Appendix Section B.2.

[5] We also elicited (mis)perceptions of risk associated with toilet ownership, such as the belief that toilets spread infections and cause sickness. Since we find no systematic differences by community wealth along these dimensions, we do not discuss them further.

[6] With respect to CLTS, Kar (2003) posits that women are 'one of the greatest internal forces for mobilisation and promotional activities in the villages'. Women may enjoy larger returns from private sanitation in terms of personal safety and privacy. Evidence from other health enhancing investments suggests the existence of gender-specific preferences in certain domains, such as health and children's welfare (Augsburg et al., 2021b, Khanna and

Table E1
Baseline characteristics by communities' wealth

	Wealthy	Less wealthy	P-value
Sanitation perceptions in the community			
Benefit perceptions (mean = 0, SD = 1)	0.090	-0.053	0.25
Risk perceptions (mean = 0, SD = 1)	-0.052	0.032	0.50
Social interactions in the community			
Community participation index (mean = 0, SD = 1)	-0.004	0.002	0.97
Trust in neighbours (0-None, 2-High, SD = 0.40)	0.808	0.970	0.00***
Religious fragmentation (0-Low, 1-High)	0.642	0.598	0.03**
Asset wealth inequality	0.951	0.612	0.00***
Communities' public infrastructure			
Has paved internal roads (%)	54.70	29.20	0.00***
Has a local hospital (%)	22.41	3.57	0.00***
Has a local primary school (%)	71.80	59.29	0.05**
Village leader characteristics			
Years as leader	11.27	8.97	0.07*
Completed primary school (%)	60.35	41.82	0.01***
Affiliated to a political party (%)	30.17	32.73	0.68
Communities	123	124	

Notes: All variables measured at baseline. Sample restricted to households interviewed at baseline and in all three follow-up survey waves. For a detailed description of these covariates, see Appendix B. **p-values <0.10, ** p <0.05, *** p <0.01.*

test these hypotheses using indicators for female-headed households and those with children, and find no evidence of heterogeneous CLTS impacts in either of these dimensions.

E.3 Alternative specifications of community wealth

In addition to the median and quartile splits for community wealth, presented in the main paper, we estimate heterogeneous impacts using a continuous wealth measure – with very similar results (see Table E3). Treated communities whose wealth is one standard deviation below the mean of zero display a reduction in OD of 7%, while the two coefficients level out to a close to zero impact for wealthier CLTS communities.

Das, 2016, Miller and Mobarak, 2013).

Table E2

CLTS impacts on OD by household characteristics

Dep. variable: main respondent performs OD	Household characteristic at baseline		
	Low ed. HoH	Female HoH	Any children
	(1)	(2)	(3)
CLTS x Yes	-0.06**	-0.05**	-0.05**
p-value (naive)	(0.01)	(0.03)	(0.03)
p-value (FWE corrected)	[0.08]	[0.14]	[0.14]
Difference	0.04*	0.02	0.02
p-value (naive)	(0.07)	(0.40)	(0.36)
p-value (FWE corrected)	[0.22]	[0.61]	[0.61]
Control mean (Yes)	0.46	0.47	0.46
Control mean (No)	0.53	0.50	0.52
Communities	246	246	246
Observations	12,697	12,697	12,697

Notes: 'Difference' is the coefficient γ_d from estimating equation 2, indicating the difference in treatment effects along heterogeneity dimensions indicated in column headings. Control means are calculated using endline data. Household controls are: age, gender, education attainment and employment status of the household head, household size, whether the household has at least one child below age 6, household wealth asset score, and whether farming is the household's main economic activity. Standard errors are clustered at the community level. Naive (unadjusted) *p*-values shown in parenthesis. In brackets we present *p*-values adjusted by family wise error rate following Romano and Wolf (2005), using 1,000 cluster bootstrap samples. **p <0.10, ** p <0.05, *** p <0.01.*

Table E3

Heterogeneous CLTS impacts on OD: continuous community wealth measure

Dep. var.: main respondent performs OD	
CLTS	-0.02
	(0.14)
CLTS × Community wealth index	0.05***
	(0.00)
Number of communities	246
Number of observations	12,697

Notes: Household controls are: age, gender, education attainment and employment status of the household head, household size, whether the household has at least one child below age 6, household wealth asset score, and whether farming is the household's main economic activity. Standard errors are clustered at the community level. *p*-values shown in parentheses. **p <0.10, ** p <0.05, *** p <0.01.*

F Community wealth measurement in developing countries

A community wealth index similar to the one in our study sample is widely available in the Demographic and Health Surveys (DHS), nationally representative samples conducted in 90 developing countries. Using the 2013 Nigerian DHS, we show that a community wealth index can be easily constructed for a national CLTS targeting strategy, and that its precision is similar to that of the more detailed measure used in our study data.

We first construct a new, comparable asset wealth index (the 'DHS index') in both samples, our study and the DHS. It is based on the joint subset of durable asset items recorded in both surveys.[7] To ensure national representativeness, we construct the index by performing a principal component analysis of the questions on the DHS sample only. The resulting index in our study data is created by applying the estimated factor loadings to households' responses.

In our study sample, the simplified DHS index, based on factor loadings from the DHS sample, is closely matched with the original household wealth index (henceforth: the 'Study index'), which was based on a more comprehensive list of asset items (see Table B1). Figure F1 shows that the two measures are highly positively correlated (ρ=0.77, significant at the 1% level). The newly created DHS index explains up to 58% of the variation in the more comprehensive Study index.

To put community and household wealth in our study sample into context, we show where they fit into the Nigerian wealth distribution. Figure F2 shows the distribution of household wealth in the representative DHS sample (black line), and in communities with wealth below (above) the median in our study sample, depicted by the blue (gray) line. First, the plot reveals that our study sample does not include the Nigerian households with the lowest assets. This is not surprising since Ekiti and Enugu are located in the relatively wealthier centre-south of the country, with the asset-poorest states located in the north. Second, our sample households are mostly located close to the median of Nigerian household wealth.

For a similar comparison, we construct community wealth deciles for Nigeria, and place our

[7] Asset items elicited in both surveys are: bicycles, motorcycles (scooters, tricycles, etc), cars and trucks, refrigerators, radio, TV, bank account, telephone (mobile or fixed), improved water, improved sanitation and livestock (cattle, goats and sheep, pigs, poultry). The inclusion of livestock is motivated by the purpose of capturing farming households. Indeed, the loading factors for these four elements are negative.

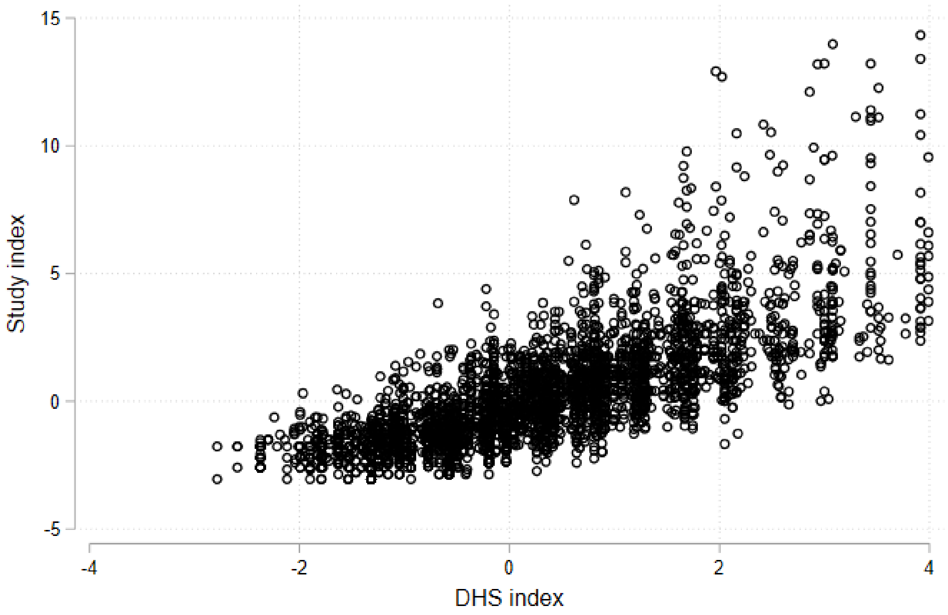

Figure F1
Study and DHS Nigeria household asset wealth indices

Notes: Scatter plot showing the asset wealth scores obtained for each household in our study sample, built using asset ownership questions from our survey (see Table B1), on the y-axis. The x-axis shows a similar index, but constructed using a list of assets elicited in both our survey and the DHS household questionnaire. Each dot represents one household.

study communities into these (see Figure F3).[8] Our study communities are typically located towards the middle (4th to 7th deciles) of the Nigerian community wealth distribution, rather than in the tails. Some study communities that we have classified as falling into the low wealth category (blue), using the median in our study, fall into the higher deciles of the Nigerian community wealth distribution. Overall, however, 80% of the less wealthy communities in our sample (blue) are in deciles 3 to 5 of the Nigerian distribution, while 88% of the communities we class as wealthy in our sample (white) are in the upper deciles of the Nigerian distribution, i.e. deciles 6 to 10. Hence, communities classified as less wealthy in our sample are generally below the median of the national distribution, too, and the majority of wealthier communities in our study are also wealthier than

[8] We calculate the median DHS index value within each DHS cluster, and within each study community. We define deciles using only the DHS sample, and use the decile cut-off values to assign study communities to the corresponding wealth deciles. Community wealth is determined using the DHS index to ensure comparability. We retain, however, the classification of less and more wealthy communities used throughout the paper, which is defined using the more comprehensive study wealth index.

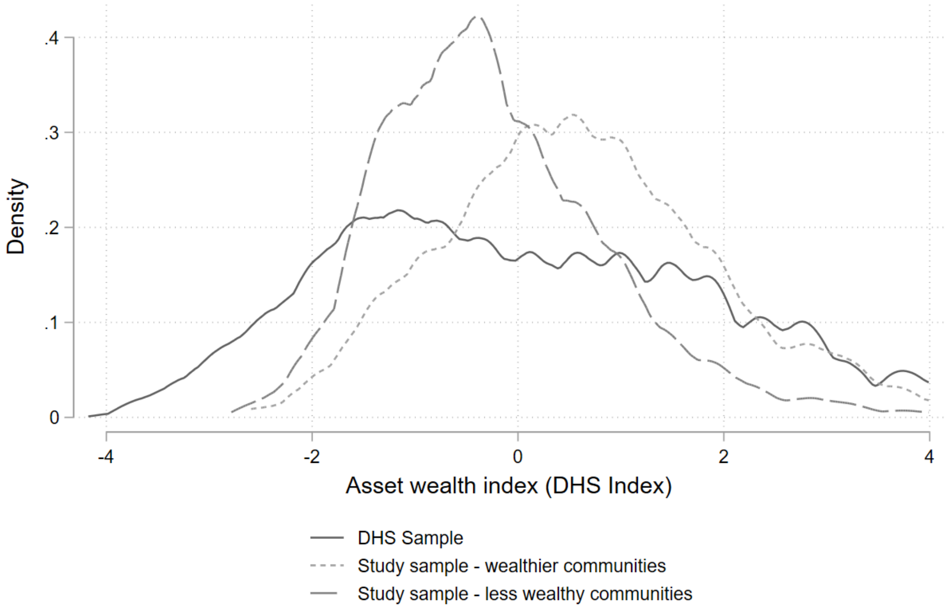

Figure F2
Distribution of the DHS Nigeria wealth index

Notes: Kernel density plot showing the distribution of wealth among the DHS sample (black), and in more (gray) and less (blue) wealthy communities in our study sample. The DHS index is the asset wealth index constructed using a list of assets elicited in both our survey and the DHS household questionnaire.

the median community in Nigeria.

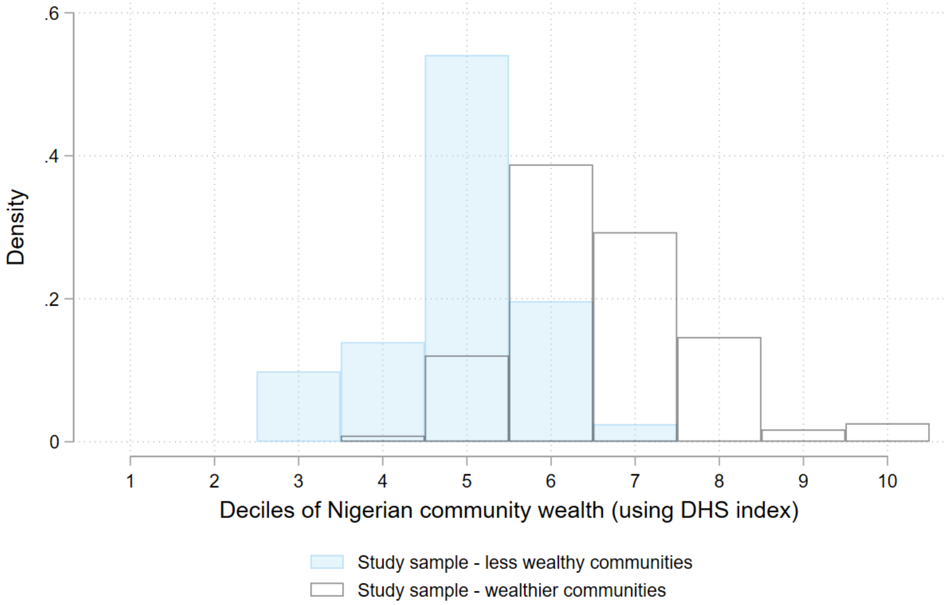

Figure F3

Distribution of study communities in DHS Nigeria community wealth deciles

Notes: Distribution of the communities in our study along community wealth deciles for the whole of Nigeria, estimated using the 2013 DHS. The wealth index used for this comparison was constructed using a list of assets elicited in both our survey and the DHS household questionnaire.

G International comparison of CLTS impacts

In the main paper, we presented evidence from pooling microdata from Indonesia, Nigeria and Tanzania and estimating average and heterogeneous CLTS impacts on toilet ownership by night light intensity.

In this appendix, we first show the robustness of our findings in Nigeria to using night light intensity instead of community asset wealth as our indicator of interest (Section G.1). We then draw on all five CLTS studies (Bangladesh, Indonesia, Mali, Nigeria, Tanzania) – which published microdata at different levels of granularity – to plot point estimates of impacts on toilet ownership and open defecation by their corresponding satellite night light intensity (Section G.2).

G.1 Night light intensity

Table G1 reproduces outcomes on toilet use and ownership, using the night light index as an alternative measure of socio-economic conditions. CLTS increases (functioning and general) toilet ownership in communities with low night light intensity but, consistent with our main findings, we find no CLTS impact in wealthier communities in terms of sanitation investments. Equally in line with the results presented in Table 3 in the main text, we find little evidence of behavioral change in terms of usage of existing toilets, or shared usage. There is weak indication that shared toilet use increases in areas that have high night light intensity. Yet, the differential impact of CLTS across community groups along shared usage is not statistically significant.

Table G1
Heterogeneous impacts by night light intensity

Outcome =1 if	Owns toilet<br>(1)	Owns functioning toilet<br>(2)	Uses toilet (if functioning)<br>(3)	Shares toilet with neighbours<br>(4)
CLTS x low night lights	0.05*	0.08***	0.04	0.00
p-value (naive)	(0.06)	(0.00)	(0.10)	(0.96)
p-value (FWE corrected)	[0.32]	[0.04]	[0.36]	[0.97]
Difference	-0.06*	-0.10***	-0.06*	0.01
p-value (naive)	(0.09)	(0.00)	(0.05)	(0.34)
p-value (FWE corrected)	[0.36]	[0.06]	[0.30]	[0.51]
Control mean (high night lights)	0.74	0.62	0.64	0.06
Control mean (low night lights)	0.53	0.40	0.68	0.07
Communities	246	246	245	246
Observations	12,497	12,497	7,113	12,697

Notes: 'Difference' is the coefficient γ_d from estimating equation 2, indicating the difference in treatment effects between communities with high and low night lights. Control means are calculated using endline data. Household controls are: age, gender, education attainment and employment status of the household head, household size, whether the household has at least one child below age 6, household wealth asset score, and whether farming is the household's main economic activity. Standard errors are clustered at the community level. Naive (unadjusted) *p*-values are shown in parentheses. In brackets we present *p*-values adjusted by family wise error rate following Romano and Wolf (2005), using 1,000 cluster bootstrap samples. *$p<0.10$, ** $p<0.05$, *** $p<0.01$.

To determine which of these measures is the stronger predictor of heterogeneous CLTS impacts, we run a horserace between the two (imperfectly) correlated proxies. Results are presented in Table G2. While the estimated coefficient on the night light interaction decreases in magnitude and is only weakly statistically significant, the asset wealth-specific CLTS impact remains statistically significant at the 5% level and does not change quantitatively. This suggests that community

wealth is a strong proxy for local socio-economic conditions in a community, and underlines our finding that CLTS is effective only in less wealthy communities.

Table G2
Heterogeneous CLTS impacts: horserace between community wealth and night light intensity

Dep. var.: main respondent performs OD	
CLTS	0.03
	(0.16)
Low asset wealth	0.07***
	(0.00)
CLTS x Low asset wealth	-0.08**
	(0.02)
Low night lights	0.07***
	(0.00)
CLTS x Low night lights	-0.06*
	(0.08)
Communities	246
Observations	12,697

Notes: Household controls are: age, gender, education attainment and employment status of the household head, household size, whether the household has at least one child below age 6, household wealth asset score, and whether farming is the household's main economic activity. Standard errors are clustered at the community level. *p*-values are shown in parentheses. *p <0.10, ** p <0.05, *** p <0.01.*

G.2 Pooling data from different contexts

We compute night light intensity at cluster level d in the studies conducted in Indonesia, Tanzania and Nigeria. We calculate average night light intensities within a 10km radius of each cluster centroid in the baseline year. We exploit within-study variation in night light intensity to calculate treatment effects separately for clusters above and below the median of each sample. For Mali and Bangladesh, detailed location information is not available. In Mali (Bangladesh), we assign the average night light intensity of the state (sub-district) in which each study took place, but do not estimate heterogeneous treatment effects.[9] Table G3 summarises night light intensities by study.

We then re-estimate heterogeneous impacts by this measure of socio-economic status for In-

[9]The Mali experiment was conducted in the state of Koulikoro, the Bangladesh experiment in the sub-district of Tanore. Columns 4 and 5 in Table G3 illustrate the high within-state variation in night light intensity around a small mean intensity, driven by Koulikoro.

TABLE G3
Summary statistics: night light intensity by study

	Level of night light extraction		Summary statistics		
Country of study	Unit (1)	N (2)	Median (3)	Mean (μ) (4)	Mean (σ) (5)
Bangladesh	Sub-district	1	0.93	0.93	2.14
Indonesia	Cluster	160	8.21	8.48	4.04
Low		81	5.69	6.07	2.86
High		79	9.98	10.96	5.25
Mali	State	1	0.13	0.13	1.59
Nigeria	Cluster	242	0.89	2.06	2.17
Low		120	0.25	0.33	1.01
High		122	2.84	3.76	3.31
Tanzania	Cluster	178	0.00	0.07	0.25
Low		167	0.00	0.01	0.13
High		11	0.00	0.13	0.39

Notes: This table shows the geographical unit at which night light data were measured in each country of study, and summary statistics. Column 1 shows the lowest administrative unit at which we could map both survey observations and location in each case. In Bangladesh, only the sub-district where the study was conducted is known (Tanore). Column 2 summarizes the available number of units identified in each study. Columns 3 and 4 estimate the median and mean respectively of night light intensity for each country. In the studies in Bangladesh and Mali, we observe night light at the sub-district level respectively for RCT clusters jointly. Column 5 shows the average within-unit standard deviation. night light intensity is truncated at zero, so higher average intensities are associated with higher within-unit variation. *Low* and *High* subsamples show the split along the within-study median value for night light intensity used in Figure G1.

donesia, Tanzania and Nigeria in a consistent manner. In Bangladesh where the experiment was conducted in a single sub-district, we estimate homogeneous CLTS impacts. We use the following specification:

$$y_{icd} = \alpha + \gamma_r T_{cd} + \theta\, y_{icd0} + X_{icd}\,\beta + \omega_d + \epsilon_{icd} \qquad (3)$$

The outcome variables, y_{icd}, are two dummy variables, denoting (i) ownership of a private, functioning toilet by household i in cluster c in district d, and (ii) OD by any member of the household. T_{cd} is a dummy variable equal to 1 if cluster c from district d has been assigned to CLTS, and 0 otherwise. We distinguish between district and cluster here, as for data protection reasons we observe treatment assignment at the anonymized cluster level c, and we observe community night

light at the non-anonymised district level d.[10] Household-level controls X_{icd} are gender, age and age squared of the household head, and whether a household's primary activity is farming. We include geographic fixed effects at the district level (ω_d) where appropriate, to account for stratified randomization.[11] Standard errors are clustered at the cluster level, the unit of randomization. For Mali, where microdata are not publicly available, we use the CLTS impact estimates reported in the published paper, obtained by a means comparison of outcomes between treatment and control households at endline (Pickering et al. (2015)).

In Figures G1 and G2 we report the resulting point estimates of CLTS impacts on toilet ownership and open defecation, respectively, by their corresponding satellite night light intensity (right axis). It illustrates that CLTS is on average more effective in settings with lower night light intensity. As night light intensity increases, point estimates decrease in magnitude for both outcomes, and the likelihood of rejecting the null hypothesis falls. Note that we find no evidence of CLTS impacts in study sites with night light intensity above that of the Nigerian median (0.89). In contrast, the three community groups with the lowest night light indices (and all samples with median night light below the less wealthy half of Nigerian communities) display statistically significant CLTS impacts. This supports the notion that CLTS seems more effective at increasing toilet ownership and at decreasing open defecation in relatively less wealthy areas.

Toilet coverage

In Table G4 we explore whether a similar result can be obtained when using community toilet coverage at the baseline for Indonesia, Nigeria and Tanzania. Columns (3) and (4) show that this measure is not a good predictor of toilet ownership, in contrast with the results we obtained using nightlight data as a proxy for economic activity or community wealth.

[10] We include baseline values y_{icd0} for the outcome variables, except for Tanzania, where no baseline data were collected. Our study in Nigeria is the only one to include multiple post-treatment survey waves, so we restricted our sample to observations from the second follow-up wave (FU2), which is closest in timing to the post-intervention surveys of the other studies.

[11] District level fixed effects are used to estimate impacts for the case of Indonesia, Nigeria and Tanzania. We omit geographical fixed effects in the case of Bangladesh since the experiment was conducted in a single district.

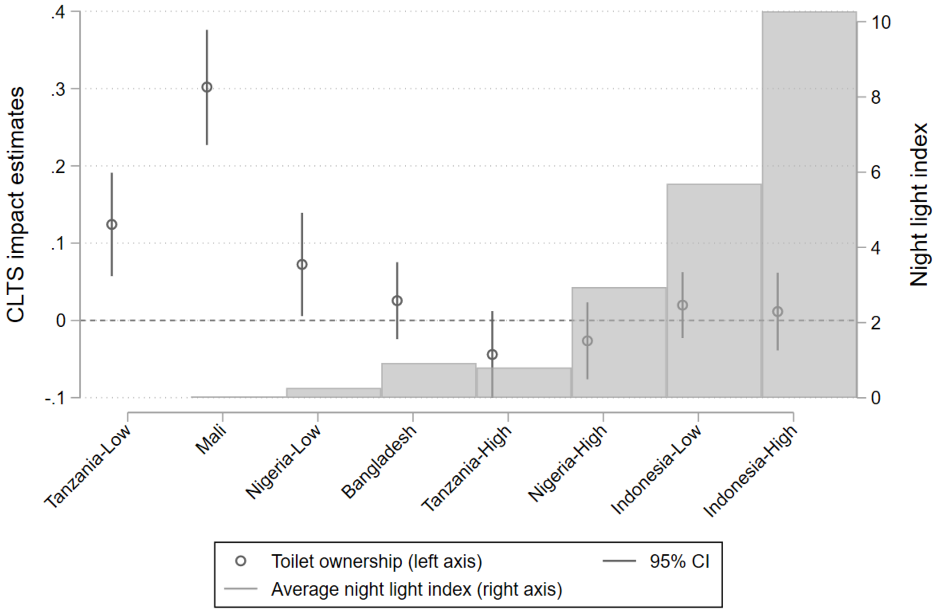

FIGURE G1

CLTS impacts toilet ownership by night light intensity

Notes: Study-specific point estimates on toilet ownership (dots). Gray bars show median night light intensity recorded by NASA's Operational Line scan system (OLS), on the year of each study's baseline survey, over the study area. Data from the Mali study were unavailable, so results from table 2 in Pickering et al. (2015) were used instead.

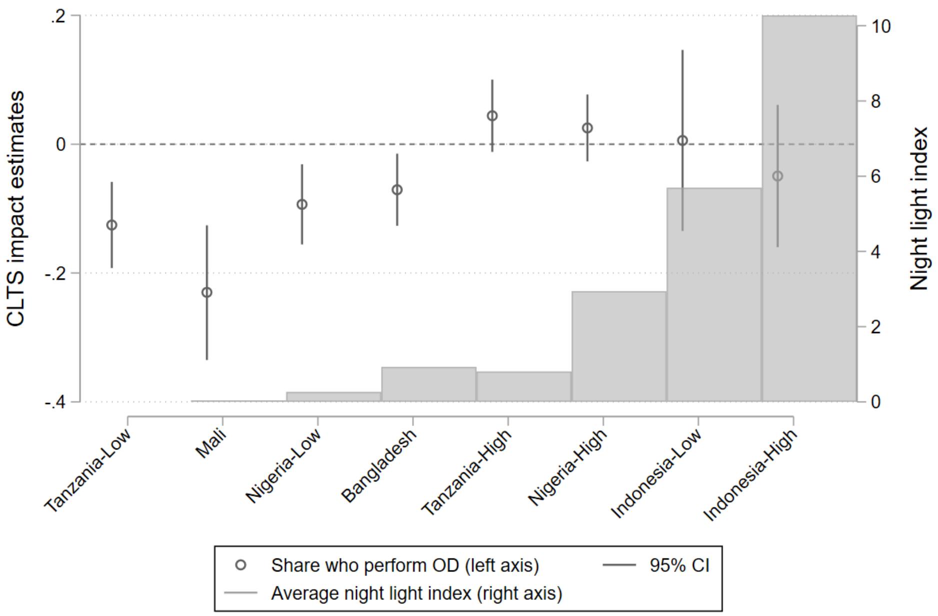

FIGURE G2
CLTS impacts on OD by night light intensity

Notes: Study-specific point estimates on OD prevalence (dots). Gray bars show median night light intensity recorded by NASA's Operational Line scan system (OLS), on the year of each study's baseline survey, over the study area. The horizontal line shows median night light intensity for Nigeria. Data from the Mali study were unavailable, so results from table 2 in Pickering et al. (2015) were used instead (results for OD for adult women were used).

Table G4
Pooled CLTS impacts by toilet coverage

Dep. variable: toilet ownership	All		By community toilet coverage (TC)	
			Below vs. above Nigerian median	TC tertiles
	(1)	(2)	(3)	(4)
Pooled average impact				
CLTS	0.05^{**}	0.05^{**}		
	(0.01)	(0.01)		
Heterogeneity by communities' toilet coverage				
CLTS in below median/1st tertile TC			0.07^{**}	0.05
			(0.03)	(0.11)
CLTS in 2nd tertile TC				0.03
				(0.37)
CLTS in above median/3rd tertile TC			0.02	-0.01
			(0.19)	(0.70)
Difference (High-Low)			-0.05	-0.06
			(0.20)	(0.13)
Difference (Middle-Low)				-0.03
				(0.54)
Country FE	No	Yes	Yes	Yes
Communities	580	580	580	494
Observations	7,843	7,843	7,843	6,988

Notes: Pooled regression results using the Indonesian, Nigerian and Tanzanian samples. All specifications control for gender, age and age squared of the household head, as well as whether farming is the main economic activity of the household. District fixed effects are also included and errors are clustered at the level of the randomization unit. p-values are shown in parentheses. **p <0.10, ** p <0.05, *** p <0.01.*
